# A Computational Model of the Ionic Currents, Ca^2+^ Dynamics and Action Potentials Underlying Contraction of Isolated Uterine Smooth Muscle

**DOI:** 10.1371/journal.pone.0018685

**Published:** 2011-04-29

**Authors:** Wing-Chiu Tong, Cecilia Y. Choi, Sanjay Karche, Arun V. Holden, Henggui Zhang, Michael J. Taggart

**Affiliations:** 1 Institute of Cellular Medicine, Newcastle University, Newcastle upon Tyne, United Kingdom; 2 Maternal and Fetal Health Research Centre, St. Mary's Hospital, University of Manchester, Manchester, United Kingdom; 3 School of Physics and Astronomy, University of Manchester, Manchester, United Kingdom; 4 Institute of Membrane and System Biology, University of Leeds, Leeds, United Kingdom; Victor Chang Cardiac Research Institute (VCCRI), Australia

## Abstract

Uterine contractions during labor are discretely regulated by rhythmic action potentials (AP) of varying duration and form that serve to determine calcium-dependent force production. We have employed a computational biology approach to develop a fuller understanding of the complexity of excitation-contraction (E-C) coupling of uterine smooth muscle cells (USMC). Our overall aim is to establish a mathematical platform of sufficient biophysical detail to quantitatively describe known uterine E-C coupling parameters and thereby inform future empirical investigations of physiological and pathophysiological mechanisms governing normal and dysfunctional labors. From published and unpublished data we construct mathematical models for fourteen ionic currents of USMCs: 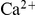 currents (L- and T-type), 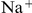 current, an hyperpolarization-activated current, three voltage-gated 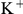 currents, two 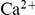-activated 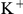 current, 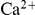-activated 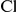 current, non-specific cation current, 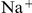-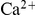 exchanger, 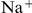-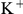 pump and background current. The magnitudes and kinetics of each current system in a spindle shaped single cell with a specified surface area∶volume ratio is described by differential equations, in terms of maximal conductances, electrochemical gradient, voltage-dependent activation/inactivation gating variables and temporal changes in intracellular 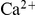 computed from known 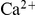 fluxes. These quantifications are validated by the reconstruction of the individual experimental ionic currents obtained under voltage-clamp. Phasic contraction is modeled in relation to the time constant of changing 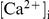. This integrated model is validated by its reconstruction of the different USMC AP configurations (spikes, plateau and bursts of spikes), the change from bursting to plateau type AP produced by estradiol and of simultaneous experimental recordings of spontaneous AP, 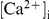 and phasic force. In summary, our advanced mathematical model provides a powerful tool to investigate the physiological ionic mechanisms underlying the genesis of uterine electrical E-C coupling of labor and parturition. This will furnish the evolution of descriptive and predictive quantitative models of myometrial electrogenesis at the whole cell and tissue levels.

## Introduction

For over 50 years it has been known that uterine smooth muscle (myometrium) generates spontaneous action potentials (APs) [1]–[3]. These precede elevations in intracellular 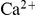 that, in turn, facilitate the actomyosin interactions governing myometrial contractions [4], [5]. The regulation of electrical activity of myometrial cells therefore plays a crucial role in determining the onset, the duration and the strength of uterine contractions during labor. This is essential for a successful conclusion to pregnancy with the safe delivery of the fetus and placenta. Unfortunately, many pregnancies result in complications of labor that compromise the health of the fetus/newborn. Preterm birth, of which activation of uterine contraction is the major cause, occurs in up to 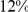 of deliveries and results in a high incidence of mortality and morbidity of the offspring [6]. Prolonged dysfunctional labor at term occurs in 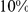 of pregnancies and these patients account for 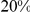 of Cesarean sections [7]. An improved understanding of the physiological complexities of myometrial electrical excitability would assist in the task of developing better targeted therapies for these problematic labors.

Modifications of myometrial cell electrophysiological characteristics during pregnancy are evident. The resting membrane potential of myometrial cells becomes progressively more positive towards term [8], gestational-dependent changes in the molecular expressions of ionic channel components occurs [9] and the form of action potentials can change between those of rapid spike-like and tonic plateau-type [10], [11]. Electrophysiological recordings have also identified several classes of individual ionic currents in myometrial cells. It is accepted that the major inward depolarizing current of the AP likely arises from 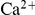 entry via L-type 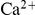 channels [12]. Other myometrial inward currents that have been suggested to be functional, at least in some experimental situations, include those mediated through T-type 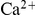 channels [13], 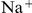 channels [14] or 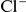 channels [15]. Voltage-dependent outward currents, both those that are sensitive or insensitive to 4-aminopyridine (4-AP), have been identified as have calcium-dependent 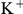 currents [16]–[20]. Molecular expression of genes/proteins of electrogenic ion exchangers, the 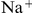-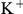 ATPase [21] and the 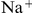-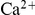 exchangers [22], suggest that these too may have a contribution to make to regulating myometrial membrane potential.

There is increasing awareness of the benefits of developing mathematical descriptions of uterine function [23]–[25] and recent attempts have shown promise regarding the mapping of electrophysiological or contractile data. However, detailed descriptions of the biophysical characteristics of each of the myometrial ionic currents are lacking. In addition, information on how these individual ionic currents are integrated to form the shape and timecourse of APs reflective of those reported for the myometrium is sparse. This severely limits the ability to model simultaneous changes in myometrial membrane potential, 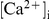 and force that are the essential elements of electrical E-C coupling. It is important to determine each of these circumstances in order to assess fully the likely physiological relevance to AP genesis of any electrophysiological data that has been recorded in isolation and attributed to a particular ion channel subtype. It is also necessary to consider how these electrical events influence E-C coupling parameters leading to the generation of phasic contractions of uterine smooth muscle as this, after all, determines the success of the parturient effort. Therefore, we had three aims to the present work. First, to develop biophysically detailed quantitative (mathematical) descriptions of all known individual ionic currents of uterine smooth muscle cells pertaining to near the end of pregnancy. Second, to compute these, in alliance with descriptions of dynamic 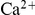 handling parameters, into a mathematical model of myometrial action potential generation. Third, to extend this model to the simulation of concomitant recordings of spontaneous AP, 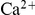 and force in uterine smooth muscle. Moreover, the model is assessed for its ability to simulate published changes in experimental parameters. The development of our quantitative model markedly advances our understanding of the electrophysiological basis of excitation-contraction coupling in uterine smooth muscle. In so doing, it also provides a framework of relevance for exploring the biophysical modeling of individual ionic currents underlying the electrogenic processes in other smooth muscles, tissues and organs.

## Results and Discussion

The general mathematical formulae used for parameter modeling are given in the Methods (equations 1–9). A glossary of symbols used in the modeling equations is given in Tables S1, S2. Detailed formulations of individual model components are given in Appendix S1 (equations 10–105).

### L-type Calcium current – 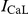


*Mathematical descriptions of the biophysical characteristics of this current are given in Appendix S1 (equations 10–19).*


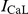
 is attributed as the major inward current in myometrial cells [8], [14], [26]–[28]. 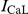 first appears at 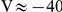 to 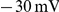; the peak of the current-voltage (I–V) relationship arises between 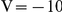 to 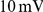 and the reversal potential 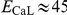 to 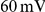 at 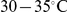 with 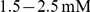 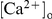 [12], [15], [29], [30]. L-type calcium channels in other cell types have been reported to be permeable to other cations [31] but there is no data specific to myometrial cells. Thus, the Goldman-Hodgkin-Katz formulation commonly used in other muscle cell models is not used here; instead, 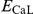 in the model is fixed at 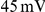 as suggested by experimental data [12], [30], [32].

**Figure 1:**
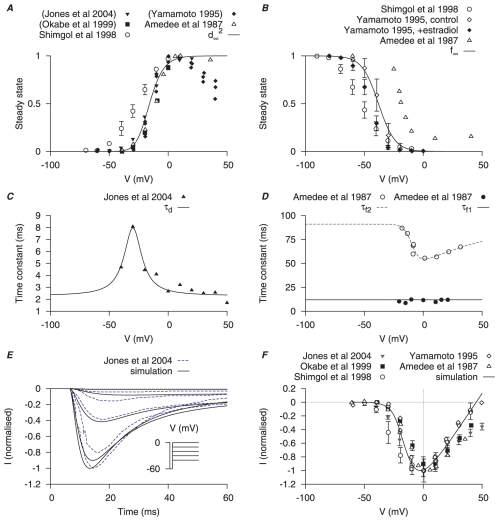
Myometrial 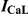 model. Properties of 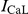 are derived from experimental data of myometrial longitudinal cells from late pregnant rat [12], [15], [29], [30], [32], [35]. *A*, voltage (V)-dependent activation steady-state (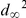); experimental data in brackets were extrapolated from current-voltage (I–V) relationships using the function 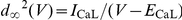 and normalized to the maximum value. *B*, V-dependent inactivation steady-state (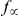). *C*, V-dependent activation time constant (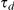); extracted by fitting current tracings from Jones *et al.* [15]. *D*, V-independent fast inactivation time constant (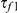, *solid circles*) and V-dependent slow inactivation time constant (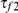, *empty circles*). *E*, simulated voltage-clamp 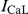 at voltage steps of 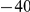 to 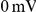 from a holding potential of 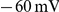 are superimposed on experimental current tracings from Jones *et al.*, [15]; *F*, simulated peak *I–V* relationship of 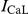 together with different experimental I–V data. In both *E* and *F*, all data are normalized to the peak current value at 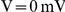.

Properties of 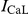 are derived from experimental data at 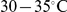 of myometrial cells from late pregnant rat. The equations of 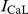 incorporate an activation gating variable (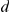) and fast (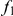) and slow (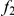) inactivation gating variables. Different steady-state values for activation and inactivation at 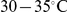 have been reported and representatives of the data range are plotted in Figure 1A–B. This may reflect different 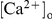 employed between studies or slightly differing residual hormonal influences. Yoshino *et al.*, [33] showed that the half-activation and the I–V relationship were right-shifted by 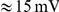 when 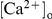 was increased from 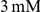 to 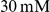; the rather rightward steady-state inactivation values from Amedee *et al.*, [29] were recorded from myometrial cells exposed to 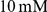 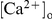. Yamamoto [30] showed that the 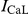 half-inactivation was left-shifted, and the I–V relationship was reduced, in the myometrial cells exposed to estradiol; in rodents, estradiol increases near term. The myometrial cells from late pregnant rats reported by Shmigol *et al.*, [12] exhibit a leftward shift in inactivation and activation curves relative to the other reports possibly reflective of an influence of altered steroidal levels near to term. Alternatively, as the holding potential (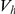) in Shmigol *et al.*, [12] was 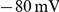, a tentative explanation could be the additional presence of 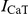 (see below) contributing to this dataset. In the model, we placed the 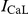 steady-state functions close to the control datasets from Yamamoto [30], which are representative of the steady-state values of 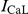 from a collection of other studies that, for clarity of presentation, are not plotted in Figure 1 [14], [33]–[35].

There is little information available for voltage-dependent activation time constants of myometrial 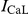, so we proceeded to extract time constants from published 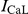 current tracings. Amedee *et al.*, [29] and Jones *et al.*, [15] had reported 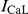 current tracings at 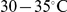, but in Amedee *et al.*, [29] only at a single voltage step and of poor quality for curve fitting purposes. There are other 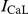 current tracings [14], [33]–[35] at room temperature but we are unaware of published 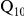 values for myometrial 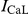. The experiments of Jones *et al.*, [15], performed at 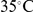, were designed to study 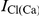 wherein 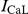 was first activated to enable plasmalemmal 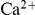 entry that, subsequently, activated a current taken to be 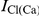. The initial fast inward current was attributed as 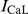 because it was blocked by nifedipine, was permeable to 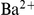 and was increased by the L-type Ca channel agonist Bay K8644. We presumed that activation of 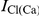 would be slower than 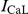 and, thus, voltage-dependent activation time constants for 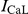 were obtained by fitting the initial few tens of milliseconds of raw data tracings, *i.e.* prior to peak current at each voltage step being reached, from Jones *et al.*, [15] (Figure 1C). This assumption is backed up by the activation time constants for 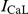 in other smooth muscles being 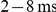 whereas that for 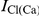 has been estimated at 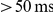 [36]. The two inactivation time constants, 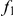 and 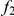, were taken from Amedee *et al.*, [29] (Figure 1D). The fast inactivation 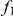 is voltage-independent at 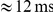 and the slow inactivation is voltage-dependent with a minimum of 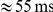 at 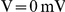.

Simulated time tracings of 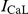 under voltage-clamp conditions and 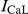 I–V relationships were compared to experimental data in Figure 1E–F. The simulated time tracings closely matched the experimental time data from Jones *et al.*, [15]; 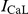 reached its peak in 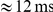 then quickly inactivated. Only the time tracings at voltage steps between 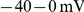 from Jones *et al.*, [15] were used for comparison in order to minimize contamination by 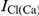. The simulated I–V relationship further shows that 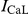 first appears at 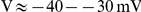 and peaks at 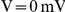, similar to that seen experimentally [12], [15], [29], [30]. Validation of the model is also evinced by the ability to reproduce the effects of estradiol on the 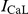 I–V relationships reported by Yamamoto [30]. Herein, the effect on the simulated I–V relationship of experimentally observed estradiol-induced changes in current were examined. The model reproduced the estradiol-mediated leftward shift in inactivation, and the reduction in I–V amplitude, from a 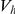 of 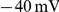 (Figure S1).

Peak 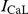 currents in myometrial cells of late pregnant rat have been reported to be 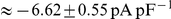 (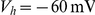, Jones *et al.*, [15]) and 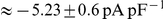 (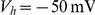, Okabe *et al.*, [32]) at 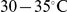. This gives a maximal conductance (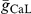) of 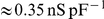 for modeling the ionic current data.

With 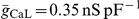 in the later development of the USMC action potential simulations, the rate of rise of an AP was 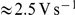 which was less than the reported experimental range of 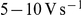 [37]. Thus, it is necessary to set 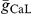 at a higher value at 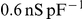.

It is possible that the reported 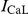 current density may represent the lower limits in late pregnant rat myometrial cells given that (i) the expression of mRNA encoding L-type Ca channel protein subunits increases before labor in rat myometrial cells [38]–[40] and the protein expression of the pore forming 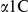 subunit is regulated by ratio of sex hormones [41]; (ii) the 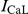 current density may be underestimated by *in vitro* experimental conditions: 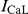 current density in isolated cells diminishes with time [11], [15]. Myometrial 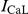 also showed calcium-dependent inactivation [26], [29]. This is described by a Hill equation with 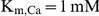 and a Hill coefficient of 4 in the whole USMC cell model.

### Sodium current – 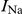


*Mathematical descriptions of the biophysical characteristics of this current are given in Appendix S1 (equations 20–27).*

Modeling of 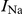 is accomplished using data from myometrial cells of late pregnant rats or humans recorded at room temperature [14], [33], [34], [42]. 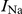 first appears at 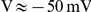 and the peak I–V relationship occurs between 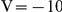 to 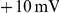. Raw data current tracings showed that 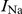 reached its peak of activation within 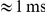 and almost completely inactivated after 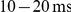 [14], [33], [34], [42].

**Figure 2:**
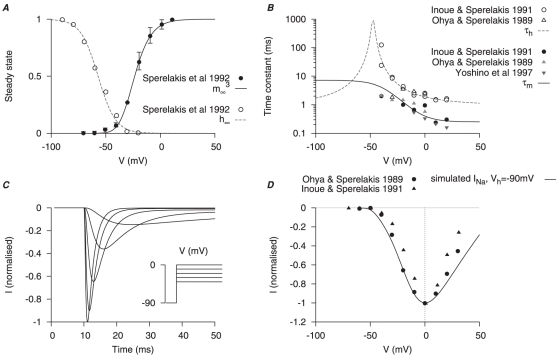
Myometrial 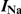 model. Properties of 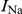 are derived from experimental data of myometrial longitudinal cells [14], [33], [34], [42] from late pregnant rats. *A*, V-dependent steady-states of activation (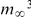) and inactivation (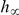); *B*, V-dependent time constants of activation (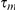) and inactivation (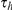). In both *A* and *B*, solid and empty circles are experimental data for activation and inactivation respectively. *C*, simulated 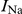 at voltage steps of 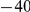 to 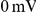 from a 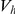 of 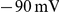; *D*, simulated peak I–V relationship of 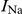 at 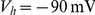 and experimental I–V data. In both *C* and *D*, all data are normalized to the peak current value at 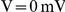.

The equation for 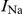 incorporates an activation gating variable (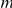) and an inactivation gating variable (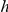). Steady-state values for activation and inactivation are shown in Figure 2A. The time constants of activation and inactivation (Figure 2B) were each obtained by fitting the raw data current tracings from the literature [14], [33], [34], [42]. Simulated traces of 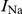 current under voltage-clamp conditions presented in Figure 2C show dynamic profiles similar to the raw data [14], [33], [34], [42]: at voltage steps of 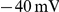 to 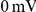, from a 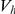 of 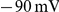, 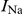 reached its peak in 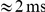 then quickly inactivated within 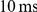. The reported peak currents for 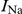 range from 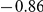 to 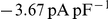 [33], [34], [42], which gives a maximal conductance range 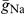 of 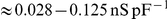. Simulated I–V relationship of 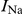 matched to the experimental data as shown in Figure 2D [14], [34], [42].

### T-type Calcium current – 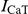


*Mathematical description of the biophysical characteristics of this current are given in Appendix S1 (equations 28–34).*


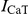
 has been reported in human myometrial cells [13], [14], [28], [37], [42]. Moreover: (i) Ohkubo *et al.*, [40] showed that the expressions of mRNA encoding for the 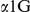 and 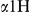 protein subunits of the T-type calcium channel were gestationally regulated in rat myometrial cells; (ii) detailed electrophysiological data of cells expressing rat 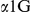/Cav3.1 are available [43], [44]; and (iii) spontaneous contractions in myometrial tissue strips from late pregnant rats were markedly inhibited by the putative T-type calcium channel blockers mibefradil, NNC 55-0396 (a non-hydrolyzable analogue of mibefradil) and 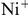 [45], [46]. Therefore, we developed a model of 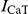 electrophysiological characteristics from the rat 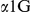/Cav3.1 clonal expression cell data recorded at room temperature [43], [44] adjusted to the current density of human myometrial cell 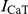 [13], [18], [28]. It is note-worthy that the activation and inactivation steady-state values, and the I–V relationships, are similar between these different datasets.


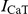
 first appears at 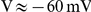, the peak I–V relationship occurs between 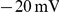 and 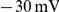, and published raw data current tracings indicate a fast activation but with inactivation temporal profiles varying between 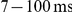 [13], [18], [28], [43], [44], [47, ] Figure S2. This last may be influenced by the different external divalent cation concentrations used between experimental conditions (Figure S3). The datasets with the fastest inactivation profiles expected of 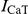 had the highest divalent cation concentrations and, indeed, were those attributed to Serrano *et al.*, [43], Hering *et al.*, [44] and Blanks *et al.*, [13].

**Figure 3:**
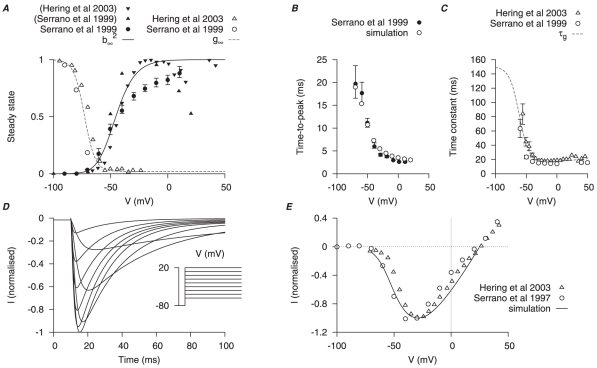
Myometrial 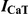 model. Properties of 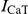 are derived primarily from experimental data of Serrano *et al.*, [43] and Hering *et al.*, [44]. *A*, V-dependent steady-states of activation (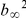) and inactivation (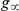); experimental data in brackets were extrapolated from the published I–V relationships and normalized to the maximum value. *B*, superimposed simulated and experimental time-to-peak of 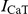 at different V stepped from 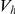 of 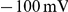; a function for the V-dependent activation time constant is chosen so that the simulated time-to-peak (*empty circles*) matched the experimental data (*solid circle*). *C*, V-dependent inactivation time constant (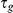). *D*, simulated 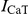 at voltage steps of 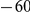 to 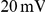 from a 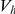 of 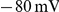; *E*, simulated peak I–V relationship of 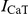 and experimental I–V data. In both *D* and *E*, all data are normalized to the peak current value at 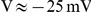.

The equation for 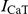 incorporates an activation gating variable (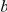) and an inactivation gating variable (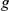). Steady-state values for activation and inactivation are shown in Figure 3A. A function is chosen for activation time constants to fit the time-to-peak experimental data (Figure 3B). The time constant of inactivation is shown in Figure 3C. Simulated 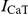 tracings under voltage-clamp conditions and I–V relationships are shown in Figure 3D and Figure 3E respectively and are compared to experimental data from Serrano *et al.*, [43] and Hering *et al.*, [44]. In Figure 3E, 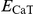 is fixed at 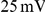 to match the experimental values in Serrano *et al.*, [43] and Hering *et al.*, [44]. The reported peak current for 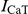 is 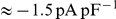 at 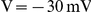 from a 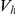 of 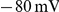 in human myometrial cells [13], which gives a maximal conductance 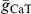 of 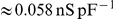. For incorporation of the 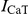 model in the later development of the USMC AP simulations, 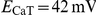 so as to mimic that of Blanks *et al.*, [13].

### Hyperpolarization-activated current – 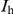


**Figure 4:**
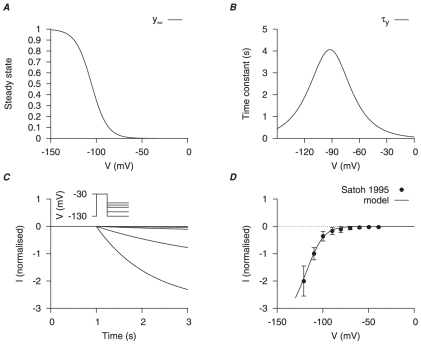
Myometrial 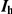 model. Properties of 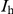 are derived from experimental data of Okabe *et al.*, [49] in rat circular myometrial cells and adjusted to experimental data of longitudinal cells [48]. *A*, V-dependent activation steady-state (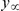); *B*, V-dependent activation time constant (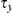). *C*, simulated voltage-clamp 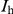 at voltage steps of 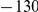 to 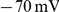 from a holding potential of 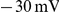. *D*, simulated I–V relationship of 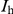 and experimental I–V data Satoh [48]. In both *C* and *D*, all data are normalized to the current value at 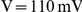.

*Mathematical description of the biophysical characteristics of this current are given in Appendix S1 (equations 35–39).*


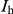
 has been reported in myometrial cells of pregnant rats [48], [49]. Activated by hyperpolarization beyond resting membrane potential, 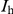 first appears at 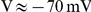 from a 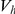 of 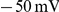. In the voltage-clamp experiments, activation of 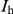 is slow, taking 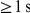, and it does not inactivate. It is more permeable to 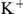 ions than 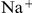 ions, is blocked by 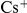, and has a reversal potential (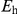) of 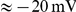.


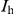
 was modeled at room temperature to 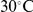 using myometrial cells of pregnant rats [48], [49]. Our model of 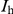 biophysical characteristics was first developed with the data of [49] with an activation gating variable (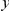) and 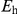 approximated by the Goldman-Hodgkin-Katz (GHK) equation with a permeability ratio 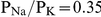. The half-activation was adjusted and the activation time constant was corrected with the reported 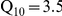 [49] in order to match the experimental I–V relationship of Satoh [48] (Figure 4). The current density was 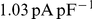 at 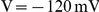 from a 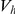 of 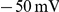 [48], which gives a maximum conductance of 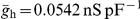.

### Potassium Currents

We have considered the electrophysiological data of several major types of potassium currents described from myometrial cells of rat and human myometrium: (two) voltage-gated potassium currents (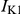 and 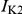), A-type transient potassium current (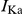) and 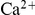-activated potassium currents (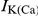). The kinetics of individual potassium currents are described in detail below; their current densities are discussed in the later section concerned with total potassium current.

### Voltage-dependent potassium currents – 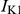 and 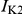


*Mathematical descriptions of the biophysical characteristics of these currents are given in Appendix S1 (equations 40–58).*

Myometrial potassium currents have been roughly categorized by their inactivation properties and sensitivity to pharmacological blockers of varying channel subtype specificity [17], [19]. At least two different types of potassium currents with rectifying properties were found in myometrial cells of late pregnant rats [17] and humans [19]; their dynamics were very slow compared to other membrane currents in myometrial cells. These potassium currents were separated as *C1* and *C2* components of the total potassium current in Wang *et al.*, [17] and as 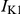 and 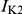 in Knock *et al.*, [19].

*C1* and 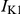, and *C2* and 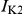 have similar voltage-dependent kinetics. Both *C1* and 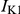 first appear at 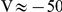 to 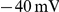 and with half-inactivation (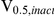) between 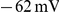 to 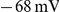. Both *C2* and 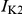 first appear at 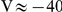 to 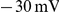 and with 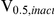 between 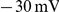 to 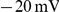. Wang *et al.*, [17] distinguished between *C1* and *C2* by their activation thresholds and inactivation properties whereas Knock *et al.*, [19] separated 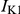 and 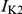 by these properties *and* current sensitivities to 4-aminopyridine (4-AP) and TEA. As such, we developed mathematical models predominantly based upon the more abundant information of electrophysiological characteristics of human myometrial 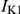 and 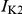 and complemented these with data on rat myometrial *C1* and *C2* of Wang *et al.*, [17] at room temperature.

**Figure 5:**
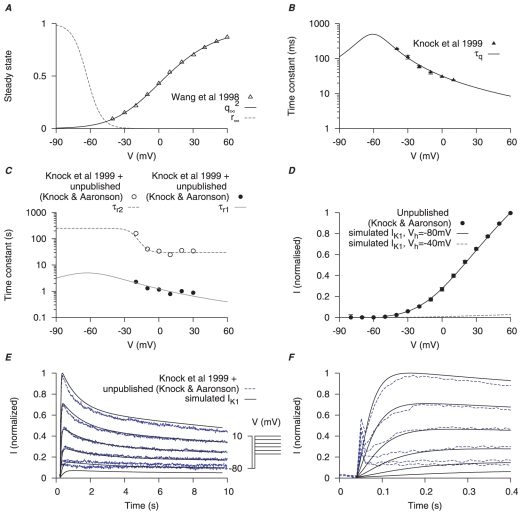
Myometrial 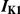 model. Steady-state properties of 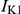 are derived from experimental data of myometrial longitudinal cells in late pregnant rats [17]; the kinetics are from myometrial cells in late pregnant women from Knock *et al.*, [19] and Knock G & Aaronson P (personal communication, including unpublished time tracings - see Figure S4). *A*, V-dependent steady-states of activation (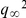) and inactivation (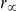). *B*, V-dependent activation time constants (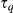). *C*, V-dependent fast (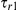) and slow (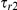) inactivation time constants. The experimental fast (*solid circles*) and slow (*empty circles*) inactivation time constants were extracted by fitting voltage-clamp time tracings averaged from five cells (1 published and 4 unpublished with the average values labeled as ‘Knock et al 1999+unpublished (Knock & Aaronson)’ in the figure). *D*, simulated I–V relationship of 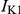 from holding potentials of 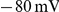 and 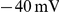 with 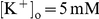 and 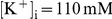; all values are normalized to the peak current at 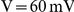 from 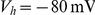. *E*, simulated time tracings and averaged raw data of 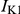 at voltage steps of 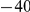 to 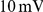 from 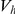 of 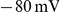; both simulated and experimental currents are normalized to the peak current at 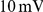; *F*, enlarged *E* showing activation of 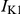 during the first few hundred milli-seconds.

**Figure 6:**
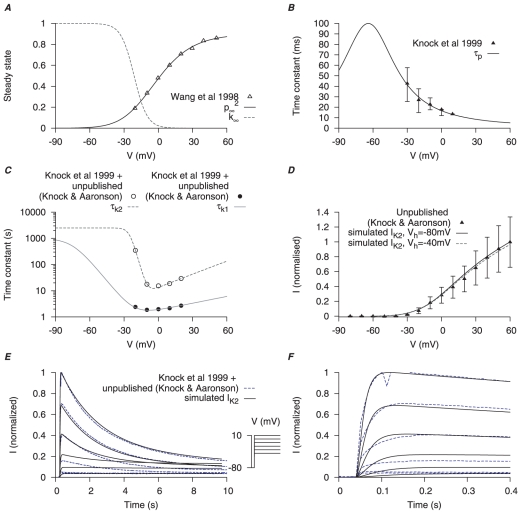
Myometrial 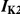 model. Steady-state properties of 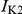 are derived from experimental data of myometrial longitudinal cells in late pregnant rats [17]; the kinetics are extracted from raw data tracings from myometrial cells of late pregnant women from Knock *et al.*, [19] and Knock G & Aaronson P (personal communication, including unpublished time tracings - see Figure S5). *A*, V-dependent steady-states of activation (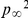) and inactivation (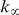). *B*, V-dependent activation time constants (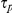) *C*, V-dependent fast (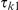) and slow (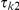) inactivation time constants. The experimental fast (*solid circles*) and slow (*empty circles*) inactivation time constants were extracted from voltage-clamp time tracings averaged from four cells (1 published and 3 unpublished with the average values labeled as ‘Knock et al 1999+unpublished (Knock & Aaronson)’ in the figure. *D*, simulated I–V relationship of 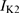 from a holding potential of 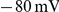 and 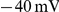 with 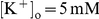 and 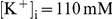; all values are normalized to the peak current at 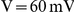 from 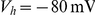. *E*, simulated time tracings of 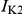 at voltage steps of 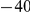 to 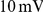 from a holding potential of 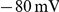; both simulated and experimental currents are normalized to the peak current at 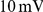; *F*, enlarged *E* showing activation of 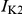 during the first few hundred milli-seconds.

The equations of 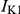 (not to be confused with the myocardial inward rectifying potassium current commonly designated also as 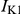 [50]) and 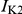 each incorporate three gating variables: an activation gating variable (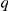 for 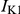; 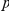 for 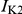), a fast inactivation gating variable (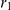 for 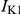; 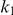 for 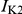) and a slow inactivation gating variable (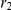 for 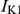; 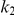 for 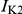). The activation and inactivation steady-state values were used as reported from Wang *et al.*, [17] with the assumption that both currents were completely inactivated (Figure 5A, 6A, see below). For 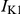, voltage-dependent steady-state of inactivation (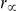) is formulated with the reported half-inactivation of 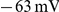 and slope factor of 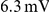 and, for 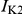, voltage-dependent steady-state of inactivation (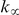) is assessed with the reported half-inactivation of 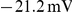) and slope factor of 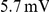 reported by Wang *et al.*, [17].

Activation time constants of 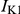 and 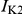 currents were from Knock *et al.*, [19] (Figure 5B, 6B) for 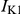 and 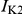 respectively. However, Knock *et al.*, [19] reported the inactivation time constants of 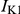 and 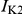 currents elicited at only one voltage step (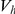 of 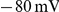 stepped to 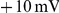): inactivation of 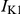 was described as a double exponential and a constant whereas inactivation of 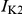 was described as a monoexponential and a constant. Their inclusion of constant values was due to the currents not inactivating during the course of the 10 sec voltage pulse. However, using these values it was impossible to simulate the published raw current tracings of the voltage-clamp protocols for 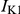 and 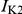 (Figure 4 in Knock *et al.*, [19]). We therefore sought to extract a more complete set of inactivation time constants that encompassed currents elicited at each voltage step of the protocols listed in Knock *et al.*, [19]. This was accomplished by examining the raw data tracings kindly supplied by Drs Greg Knock and Phil Aaronson (Kings College London). The 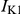 or 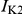 currents in each of these datasets were produced in 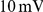 steps between 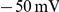 and 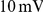 from a 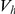 of 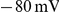. Averaging the 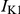 (5 cells, Figure S4) or 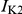 (4 cells, Figure S5) at each step enabled a calculation of the voltage-dependent inactivation time constants (Figure 5C and 6C for 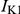 and 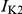 respectively). The inactivations of 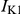 and 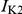 were described by a fast and a slow time constants. Moreover, we removed the need for a constant value used by Knock *et al.*, [19] by assuming that each current was completely inactivated. This, in fact, was reported to be the case by Knock *et al.*, [19] when they extended the experimental voltage pulses beyond 10 seconds. Satisfactory simulation of the published I–V curves and raw current data was now possible. Simulated I–V relationships of 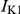 and 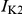 (Figure 5D, 6D) stepping from two different 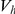, 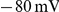 and 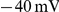, showed that while 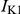 was mostly inactivated with 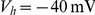, 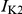 remained available. From the simulated current tracings (Figure 5E, 6E) both 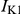 and 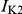 took more than 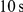 to inactivate but 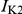 was inactivated faster than 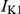. Current densities of 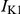 and 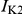 are discussed in the section of total potassium current.

### A-type transient potassium current – 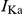


**Figure 7:**
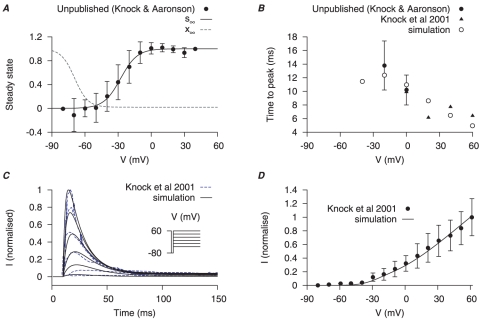
Myometrial 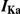 model. Properties of 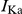 are derived from experimental data of myometrial cells from Knock *et al.*, [19], [51] and Knock G & Aaronson P (unpublished data, personal communication) in late pregnant women. Functions for V-dependent activation and inactivation time constants are chosen so that the simulated time-to-peak, current tracings and I–V relationship matched the experimental data. *A*, V-dependent steady-states of activation (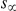) and inactivation (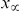). *B*, simulated (*empty points*) and experimental (*solid points*) time-to-peak of 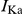 at different V stepped from a 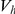 of 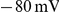. *C*, simulated voltage-clamp 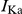 at voltage steps of 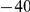 to 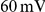 from a holding potential of 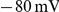 are superimposed on experimental current tracings from Knock *et al.*, [51]; *F*, simulated peak I–V relationship of 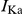 and experimental I–V data. In both *E* and *F*, all data are normalized to the peak current value at 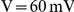.

*Mathematical descriptions of the biophysical characteristics of this current are given in Appendix S1 (equations 59–65).*


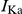
 is a 4-AP sensitive, TEA-insensitive potassium current with very fast activation and inactivation kinetics. It is found in myometrial cells of both rat and human [27], [51].


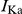
 is first evident at 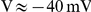 and raw data tracings show 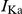 peak activation within 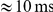 and almost completely inactivated within 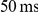 [27], [51]. In human myometrial cells, 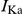 has a half-inactivation of 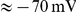 and a slope factor of 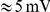 [19], [51]. These characteristics are very similar to the transient potassium current in myometrial cells isolated from immature rats [52] which were inhibited by 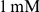 of 4-AP and were measured within 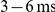 of the voltage step; it has a half-inactivation of 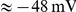 and a slope factor of 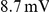.


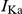
 is modeled from data of myometrial cells from pregnant rats and humans recorded at room temperature. The model of 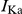 incorporates one activation gating variable (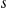) and an inactivation gating variable (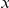). Steady-state values for activation and inactivation are shown in Figure 7A. Voltage-dependent steady-state of inactivation 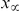 is formulated with the reported half-inactivation of 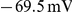 and slope factor of 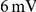 reported by Knock *et al.*, [19]. The activation time constants were chosen to fit the time-to-peak experimental data (Figure 7B). Experimental values of steady-state and time-to-peak are kindly provided by Drs Greg Knock and Phil Aaronson (Kings College London). The inactivation time constants were obtained by fitting the raw data current tracings from Knock *et al.*, [51] and the simulated time tracings showed dynamics similar to the experimental time tracings (Figure 7C). The simulated I–V relationship shows that 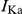 first appears at 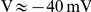, similar to experimental data [51] (Figure 7D). Current density of 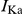 is discussed in the section of total potassium current.

### Calcium-activation potassium current – 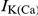


*Mathematical descriptions of the biophysical characteristics of this current are given in Appendix S1 (equations 66–78).*

Calcium-activated potassium currents (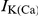) have been suggested to play important roles in suppressing the excitability of smooth muscle cells especially those in the vasculature. In myometrial cells 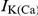 is under complex gestational-mediated regulation: the large conductance 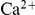-activated 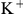 channels (termed 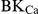 channel) subunit compositions and current density are diminished near to term. As such, although 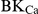 channels have been a focus of much interest in the myometrium [16], [17], [19], [53]–[62], detailed biophysical information on 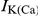 whole cell current is rather restricted.

When detected in myometrial whole cell recordings, 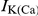 was distinctly noisy and its activation was almost instantaneous [17], [27]. From the reported recordings of 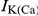 in myometrial cells by Khan *et al.*, [16], [61], [62], Wang *et al.*, [17] and Noble *et al.*, [20] many of the biophysical parameters required to model complete ion current characteristics are absent. Therefore, a biophysical quantification of the 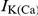 current is developed from experimental whole cell electrophysiological data obtained at room temperature from cloned mammalian smooth muscle 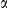 (pore-forming) and 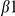 (regulatory) subunits of 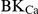 subsequently expressed in *Xenopus laevis* oocytes [63], [64]. The current densities of 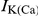 in the model are adjusted to replicate published human myometrial cell data [65], [66].

We assumed that the transmembrane 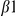 subunits were separately regulated from the pore-forming 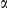 subunits and, therefore, two subtypes of 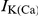 were developed: one where 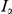 reflects an 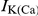 consisting of 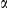 subunits; another where 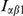 represents an 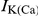 consisting of 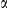 and 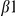 subunits; the total 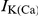 is then taken as the sum of 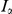 and 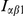. This also enabled investigation of the effects of changing voltage- and calcium-sensitivities of 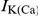.

**Figure 8:**
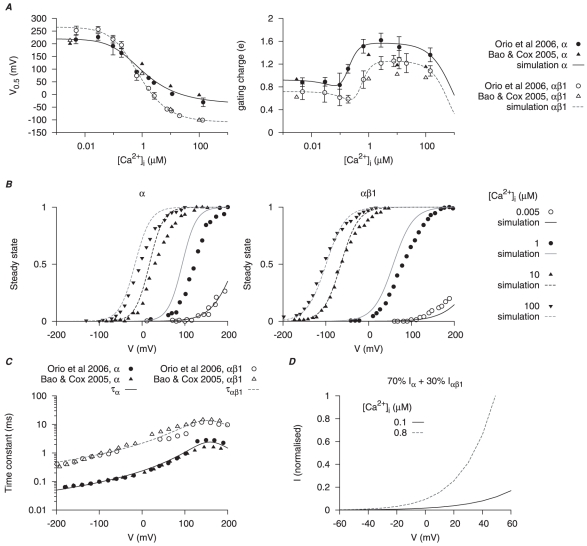
Myometrial 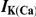 model. The calcium- (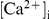), voltage- (V) and time-dependent kinetics for the two types of 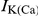 currents, 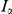 and 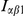, are developed with experimental data from cloned mammalian myometrial and smooth muscle MaxiK 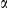 and 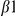 subunits expressed in *Xenopus laevis* oocytes [63], [64]; the current density and proportion of 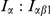 are adjusted with I–V relationships from different mammalian myometrial cells [17], [65], [66]. In *A* and *C*, solid and empty circles are experimental data for 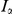 and 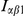 respectively. *A*, 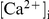-dependent half-activation (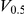) and activation gating charge. *B*, simulated activation steady-states for 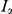 and 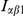 at different 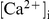; solid and empty circles are experimental data from Orio *et al.*, [64] and Bao & Cox [63] respectively. *C*, V-dependent activation time constants for 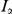 and 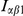. *D*, simulated I–V relationships of 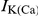 at anticipated myometrial resting and peak 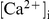 levels, with the proportion of 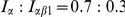. Both I–V relationships are normalized to 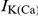 at 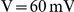 at peak 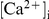 level.

The conductances of 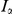 and 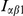 are each modeled by an activation gating variable (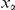 for 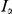; 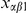 for 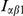). The half-activation and the corresponding gating charge were functions of 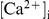 (Figure 8A); the simulated activation steady-states in comparison to the experimental values at different 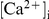 [63], [64] are shown in Figure 8B and the activation time constants in Figure 8C. A ratio of 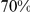 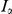 to 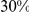 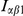 was found to produce the best fit of myometrial cell experimental I–V relationships [65], [66]. Using estimates of resting and peak global 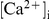 in myometrial cells of 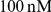 and 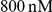 respectively [67], the simulated I–V curves showed that high 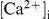 increased 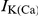 at positive membrane potentials (Figure 8D). Current density of 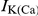 is discussed in the section of total potassium current.

### Background potassium current – 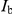


*Mathematical description of the biophysical characteristics of this current are given in Appendix S1 (equation 79).*

We have described so far the biophysical properties of the major myometrial 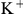 currents for which there is sufficient detailed electrophysiological information (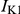, 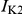, 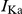 and 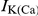). Other, less biophysically detailed electrophysiological information, together with evolving molecular and pharmacological data, suggests the possible existence of other myometrial 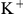 current sub-types including small-conductance 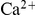-activated 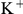 channels (termed 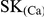) and voltage-dependent Kv7 (KCNQ) channels [20], [68]–[71]. Therefore, 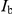, a linear background potassium current is added and it collectively represents the remaining 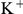 currents.

### Whole cell total potassium current – 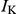


In order to model the whole cell 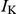 it is necessary to combine the current densities of each of the potassium current components.

The current densities of voltage-gated potassium currents (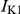 and 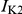) reported in myometrial cells show considerable variability. The total voltage-gated potassium current at the voltage step of 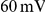, from 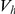 between 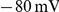 and 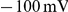 in myometrial cells studied by Knock *et al.*, [19], [51] varied between 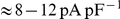. Interestingly, the majority of human myometrial cells consisted of either 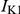 (24/42 cells) or 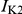 (18/42 cells) as the dominant potassium current [19] with only a very small number of myometrial cells reported to exhibit both 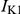 and 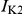 [51]. In contrast, Wang *et al.*, [17] reported a voltage-gated potassium current density of 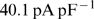 at 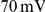 from 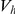 of 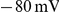. The potassium current was a mixture of 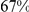 *C1* (corresponding to 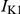 in Knock *et al.*, [19]) and 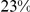 *C2* (corresponding to 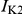 in Knock *et al.*, [19]) and, together, they accounted for almost 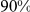 of total potassium current during a 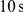 voltage step; the remaining 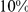 were sustained currents consisting of mostly 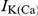 with an activation threshold of 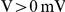.

**Figure 9:**
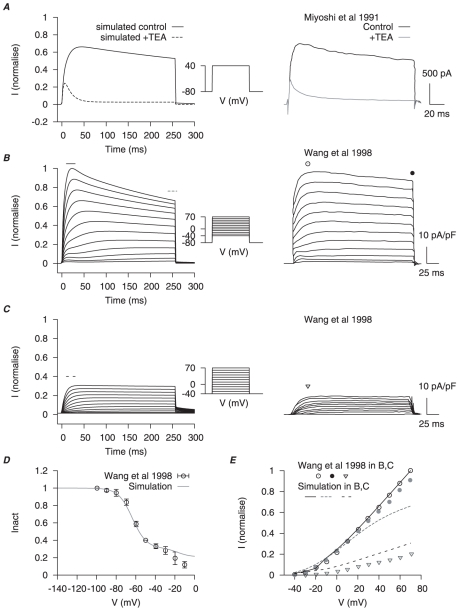
Myometrial total 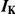 model. Potassium currents including 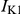, 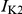, 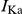, 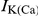 and 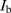 were combined to simulate the whole cell 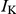 data of Miyoshi *et al.*, [27] and Wang *et al.*, [17]. *A*, simulated effects of 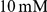 TEA (*left*), which blocks 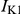, 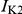 and 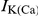 but not 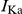, at a voltage step of 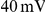 from a holding potential (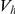) of 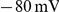; corresponding experimental results [27] (*right*). *B*, simulated whole cell potassium currents (*left*) and corresponding experimental results [17] (*right*) at voltage steps from 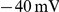 to 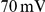 from a 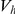 of 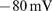; and *C*, from a 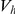 of 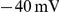. *D*, simulated inactivation of whole cell potassium currents with the same two-step protocol in Wang *et al.*, [17]: 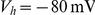, followed with a 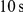 conditional step ranging from 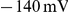 to 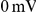, then a final test step at 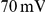 for 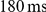. The peak current during the the test steps is normalized to test step at 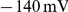. *E*, the I–V relationships at peak and at the end of the voltage step in *B* and *C*. In *B* and *C*, simulated currents are normalized to the peak current at 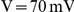 from 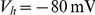.

The reported peak current for 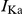 ranges between 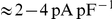 in human myometrial cells [51] and 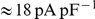 in rat myometrial cells [27] at voltage steps of 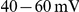 from a 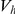 of 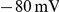. However, from the raw time tracing [27], [51], the ratio of the peak 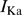 (occurring at 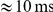) with respect to the peak total potassium current (occurring at 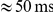) was consistent at 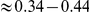 over a range of voltage steps from 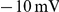 to 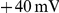. Therefore, the maximal conductance of 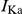 was chosen so that the peak of 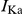 corresponds to 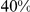 of the peak total potassium current (Figure 9A).

We have chosen the maximal conductances of 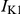, 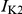, 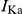, 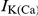 and 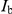 such that, together, the simulated total potassium current under different voltage-clamp protocols fits the profiles of experimental voltage-clamp results in Miyoshi *et al.*, [27] and Wang *et al.*, [17] (Figure 9).

In the later development of the USMC AP simulations, the total potassium current density was scaled to match the experimental data of whole cell potassium current in Okabe *et al.*, [32]; 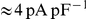 at 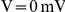 from a 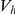 of 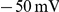.

### Other membrane currents

A non-selective cation current (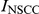) and a calcium-activated chloride current (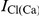) have been reported for myometrial cells from late pregnant rats. We also formulated electrogenic currents for the 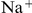-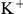 ATPase and 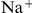-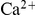 exchangers, 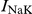 and 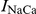 respectively, by extrapolating data from other cell systems. 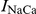 will be discussed with 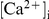 dynamics in a later section.

### Calcium-activated chloride current – 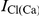


*Mathematical descriptions of the biophysical characteristics of this current are given in Appendix S1 (equations 80–86).*

The presence of channels permeable to chloride in myometrial cells was first reported by Coleman & Parkington [72]. Subsequently, there have been several reports of calcium-activated chloride current in myometrial cells, albeit the biophysical characteristics have not been as thoroughly explored as in other smooth muscles and tissues [15], [17], [73], [74]. In addition, Clca isoforms 3 and 4, suggested to encode for channel proteins responsible for 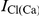, have been found in the uterus and the induced expression of Clca4 in mammalian cells elicited a calcium-dependent chloride current [75], [76].

**Figure 10:**
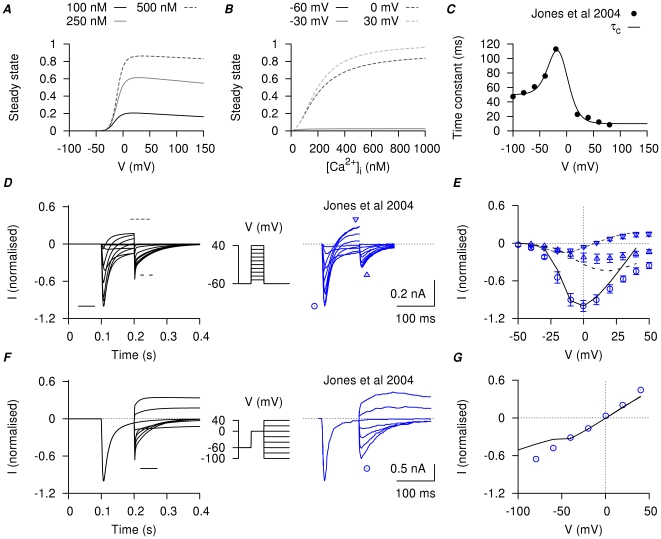
Myometrial 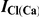 model. The steady-state of 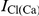 is modified from Arreola *et al.*, [77]. *A*, steady-state of 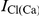 with respect to V in three different 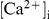 concentrations; *B*, steady-state of 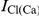 with respect to 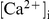 at four different membrane potentials. *C*, V-dependent activation time constant; the experimental data points are obtained by fitting the tail currents in figure 2 of Jones *et al.*, [15]. *D*, simulated currents (*left*) and the corresponding experimental currents in Jones *et al.*, [15] (*right*) elicited by a single-step voltage-clamp protocol (*inset*). The peak of the inward currents, the current values at the end of the voltage pulse, and the peak of the tail currents were marked for both simulated current (*lines*) and experimental current tracings (*circles*). *E*, I–V relationships, showing the marked peak at each voltage step in *D*. *F*, simulated currents (*left*) and the corresponding experimental currents in Jones *et al.*, [15] (*right*) by a two-step voltage-clamp protocol (*inset*). The peak of the tail currents were marked for both simulated current (*lines*) and experimental current tracings (*circles*). *G*, I–V relationships, showing the marked peaks of the tail currents at each voltage step in *F*. The simulated currents qualitatively reproduced the experimental current tracings in both voltage-clamp protocols, with almost zero net current at the holding potential and comparable amplitude and rate of decay of the tail currents.

The only serious single cell electrophysiological assessment of 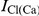 in myometrial cells (rat, 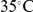) is from Jones *et al.*, [15] and therefore, this is the experimental data used for our modeling purposes. They used two different voltage-clamp protocols: a single step voltage-clamp and a two-step voltage-clamp (illustrated in Figures 1 and 2, respectively, of Jones *et al.*, [15]). Both protocols relied on the activation of 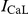 to raise 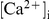 which, in turn, was proposed to activate 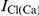. 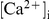, however, was not clamped in Jones *et al.*, [15] and so, for modeling purposes, it was not possible to determine the steady-state values nor the activation kinetics. However, such information is available from the data of Arreola *et al.*, [77] for 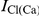 in rat parotid acinar cells whereupon 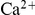 buffers were introduced intracellularly to control 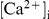. This enabled the recording and modeling of calcium- and voltage-dependencies of 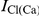. In addition, the Arreola *et al.*, [77] model could reproduce the calcium- and voltage-dependencies of 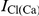 in pulmonary vascular smooth muscle cells [78]. As such, we applied the model of Arreola *et al.*, [77] to simulate the myometrial data of Jones *et al.*, [15]. Utilizing the values for the calcium-dependent time constant of activation from Arreola *et al.*, [77], or even changing them substantially, failed to provide a suitable fit to the Jones *et al.*, [15] 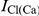 dynamics. If one assumed only a voltage-dependency to the activation time constant then the raw data time tracings of Jones *et al.*, [15] could be fitted by the Arreola *et al.*, [77] model (Figure 10). Thus we include 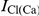 in our later model of USMC AP form with the caveat that the activation kinetics are different from that described in other cells [77], [78].

### Non-selective cation current – 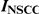


*Mathematical descriptions of the biophysical characteristics of this current are given in Appendix S1 (equations 87–92).*

Miyoshi *et al.*, [79] had identified a non-specific cation current in late pregnant rat myometrial cells. 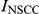 is a linear, time-independent cation current. It is permeable to 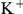, 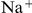, 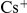 and 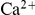, with relative permeability ratios of 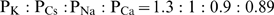. The conductance of 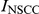 depends on extracellular concentrations of permeable cations and it was inhibited by extracellular 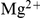, 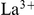 and 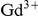. The reported reversal potential and current density under standard conditions in Miyoshi *et al.*, [79], with 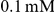 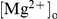 and utilizing a voltage ramp protocol, were, respectively, 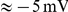 and 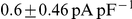.


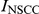
 is modeled with data from late pregnant rat myometrial cells recorded at room temperature. The reversal potential of 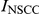 (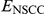) is approximated by the Goldman-Hodgkin-Katz (GHK) equation [80] with the reported permeability ratio [79]. Intracellular and extracellular concentrations of 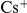 and NMDG with 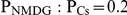 were included in the calculation of 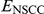 while fitting experimental data in Miyoshi *et al.*, [79]; these parameters for 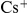 and NMDG were excluded in the later development of the USMC whole cell model.

The conductances of 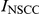 for different cations from the voltage ramp I–V relationships have a ratio of 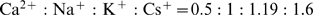 [79]. Similar to 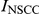 in guinea-pig endocardial endothelial cells [81], conductance of myometrial 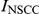 was reduced with decreasing 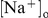. With reference to Manabe *et al.*, [81], this relationship was described by a Hill equation with a half-saturating concentration of 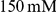 and a Hill coefficient of 2. We have normalized the Hill equation with the 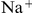 conductance at 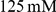 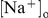 and we assumed the same relationship held for other permeable cations; for 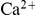 ions, the Hill equation is normalized to the 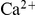 conductance observed at 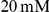 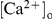. Inhibition by 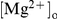 is described by a Hill equation with a half-saturating concentration of 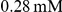 and a Hill coefficient of 1.3 [79]. 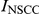 is also permeable to other cations ions [79] and, therefore, a small leak component (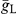) in its conductance is needed to match the experimental voltage ramp data. Under physiological conditions with 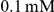 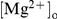 the simulated 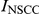 consists of mostly 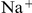 and leak components.

### Sodium potassium pump current – 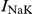


*Mathematical descriptions of the biophysical characteristics of this current are given in Appendix S1 (equations 93–96).*

Evidence of 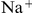-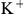 pump activity has been reported in myometrial cells of late pregnant rats [82]–[84] and human [8]. mRNA and protein expression corresponding to 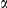 and 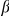 subunits of the 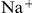-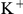 ATPase have been reported in rodent and human myometrium with isoform-specific changes associated with advancing gestation and/or estrogen treatment [21], [84]–[86]. In sodium-rich myometrial tissues of late pregnant rats [82], [83] and human [8], changes of the membrane potential were sensitive to ouabain, the absence of external potassium or intracellular sodium and to low temperature, results that are suggestive of an electrogenic 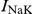. Despite this molecular and biophysical data supporting a role of the 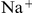-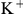 pump in regulating myometrial activity, there is little information about the biophysical properties of 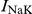 current in myometrial cells. Therefore, we adopted the formulation of an electrogenic 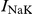 from rodent myocardial cells [87], which was dependent on membrane voltage, 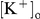, 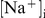 and 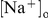. The parameter values of voltage, 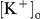 and 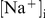 dependencies, as well as current densities, are then fitted with the experimental data from rodent vascular smooth muscle cells [88] at 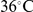. A 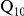 value of 1.87 for 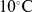 change between 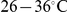 is reported for vascular smooth muscle cells [88]. We assumed the same 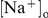 dependency with 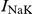 in smooth muscle cells as in the myocardial cells.

### Calcium fluxes

*Mathematical descriptions of the plasmalemmal* 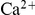 *fluxes are given in the* *Methods* *(* *equation 7* *) and Appendix S1 (equations 97–103).*

In myometrial cells from near-term pregnant rats, intracellular 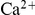 ions are removed from the cytoplasm principally by the plasmalemmal 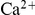-ATPase (PMCA) and 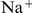-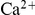 exchanger [12], [67], [89], [90]. From the decay rate constants, 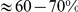 of cytoplasmic 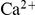 removal was estimated to be *via* the 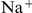-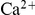 exchanger and sequestration into intracellular stores, and 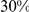 *via* PMCA when the cell was stimulated by ten short depolarization pulses between 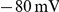 and 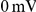 [67].

We modified a myometrial intracellular calcium model [24] for inclusion in the development of the USMC AP simulations by incorporating time-dependent kinetics from membrane calcium currents. We also modified the formulation of the 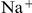-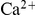 exchanger to overcome its limits in fitting published 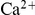 decay tracings. For example, we found that the calcium decay tracings in Shmigol *et al.*, [67] and Shmigol *et al.*, [12] could only be fitted by the procedure described in Bursztyn *et al.*, [24] with 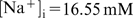. However, no sodium ions were included in the pipette (intracellular) solution used by Shmigol *et al.*, [67]. The resultant reversal potential of the 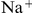-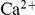 exchanger was predicted at 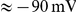 which would mean the 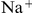-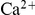 exchanger **bringing in** extracellular calcium at resting membrane potentials of 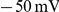 to 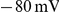 which is incorrect. Our use of the well-described formula of Weber *et al.*, [91] obviated this and enabled us to fit the 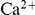 fluxes with the same ionic concentrations used in Shmigol *et al.*, [67] and Shmigol *et al.*, [12]. With 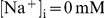, the resultant reversal potential was in the positive membrane potential range and, thus, the 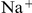-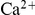 exchanger was predicted to **extrude** intracellular 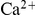 in the physiological range of resting membrane potentials.

We have modeled three major plasmalemmal calcium fluxes: the voltage-dependent membrane channels permeable to 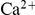 (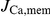); the 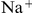-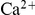 exchanger (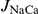); and the PMCA (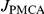).

The parameters for 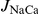 and 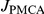 are refitted with experimental results of calcium decay in late pregnant rat myometrial cells recorded at 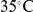 from Shmigol *et al.*, [12], [67]; the modified calcium sub-system is further validated with experimental data (Figure S7A). Details of individual fluxes are described below.

### Membrane 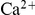 channels – 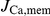



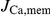
, which includes all the membrane ion channel calcium currents: 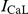, 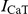 and the calcium component of 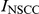 (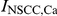), was calculated from the total membrane calcium current as described in the Methods (equation 7).

### Sodium-calcium exchanger – 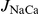


The 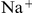-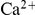 exchanger has been suggested to be involved in calcium translocation in myometrial cells from pregnant rats [12], [67], [89], [90]. However, it is unknown whether the myometrial 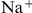-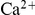 exchanger is electrogenic although the earliest studies of the effects of changing 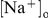 and 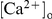 on the rat myometrial cell membrane properties suggested so [92].

There are three 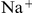-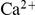 exchanger isoforms (NCX1, NCX2, NCX3) and NCX mRNA and protein has been reported in myometrium [93], [94]. NCX2 is the predominantly expressed isoform in smooth muscle tissues, including the uterus, but its stoichiometry and electrogenicity are unknown. Cloning of NCX2 [95] shows that it shares 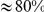 similarity in amino acid sequences with NCX1, the predominant isoform in heart tissues, and they were functionally similar with respect to their I–V relationship and voltage-dependency [96], [97]. Compared to NCX1, NCX2 has a higher dissociation rate (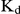) for 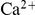 at 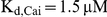 and a lower 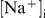 affinity at 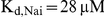. As the 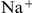-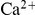 exchangers in cardiac myocytes [98] and aortic smooth muscle cells are electrogenic [99] and the properties of NCX1 and NCX2 isoforms are similar, we presumed the myometrial sodium calcium exchanger would also be electrogenic.

We used an electrogenic 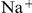-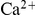 exchanger equation for cardiac cells from Weber *et al.*, [91] that describes current dependencies on membrane potential, intra- and extra-cellular calcium and sodium concentrations and has a stoichiometry of 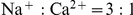. Dissociation constants for 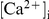 and 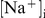 were set as 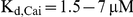 and 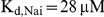, respectively [22], [95]. Dissociation constants for 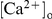 and 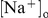 were assumed the same as Weber *et al.*, [91]. The maximum calcium flux *via* 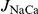 and parameters for 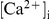 allosteric activation were refitted with experimental results of calcium decay in late pregnant rat myometrial cells [12], [67]. Membrane current from the 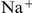-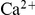 exchanger, 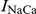, is converted from the fitted calcium fluxes 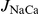.

### Plasma membrane 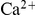 ATPase – 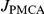


PMCA activity in rat myometrial cells has been characterized in fractionated plasma membranous vesicles with a reported ATP-dependent uptake with half saturation at 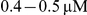 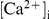 and a Hill coefficient of 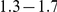 [22], [100]–[102]. PMCA is described by a Hill equation with a half saturation at 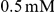 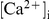 and a Hill coefficient of 2.

### Cell and tissue modeling: simulations of APs, 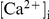 and force

Our ability to integrate the information obtained from the above biophysically detailed models of individual ionic fluxes into simulations of APs and the ensuring changes in 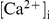 and force at a cellular/tissue level were assessed by the following validations.

### Model validation 1: simulation of different myometrial action potential configurations

Myometrial cells can produce different forms of APs including those consisting of a single spike, a burst of spikes or a plateau-type. A first task of validation was to assess if integration of our individual ionic current models and 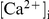 fluxes could simulate these different AP forms.

We began to assemble a model of AP configuration that incorporated all of the currents and ion fluxes described above. However, under physiological conditions of ionic concentrations [32], this model configuration produced a resting membrane potential (RMP) that was too depolarized (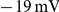) and a basal 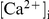 that was too high (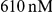). Many of the ionic currents described above were found in only a subset of the studied myometrial smooth muscle cells. In particular, 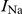 was reported in only 2/30 myometrial cells in Miyoshi *et al.*, [27]. Removing 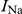 from the model, therefore, produced an RMP of 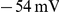 with a resting 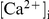 of 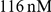. When some 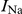 is included (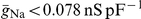), the USMC model became more excitable with lower voltage threshold (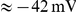) and current threshold (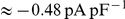, 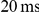 stimulus). The parameters and initial conditions of the USMC model configuration are given in Table S3, S4.

The USMC model is excitable and responds to a brief stimulus with an all-or-none AP. The voltage threshold is 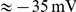; the corresponding current threshold is 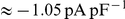 by a 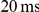 stimulus. The simulated AP usually overshoots 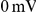 with a maximum rate of rise (dV/dt) up to 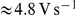 and the AP duration (APD) measured at 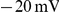 ranges between 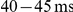, similar to the experimental values of dV/dt [37] and APD [100] for rodent myometrium.

**Figure 11:**
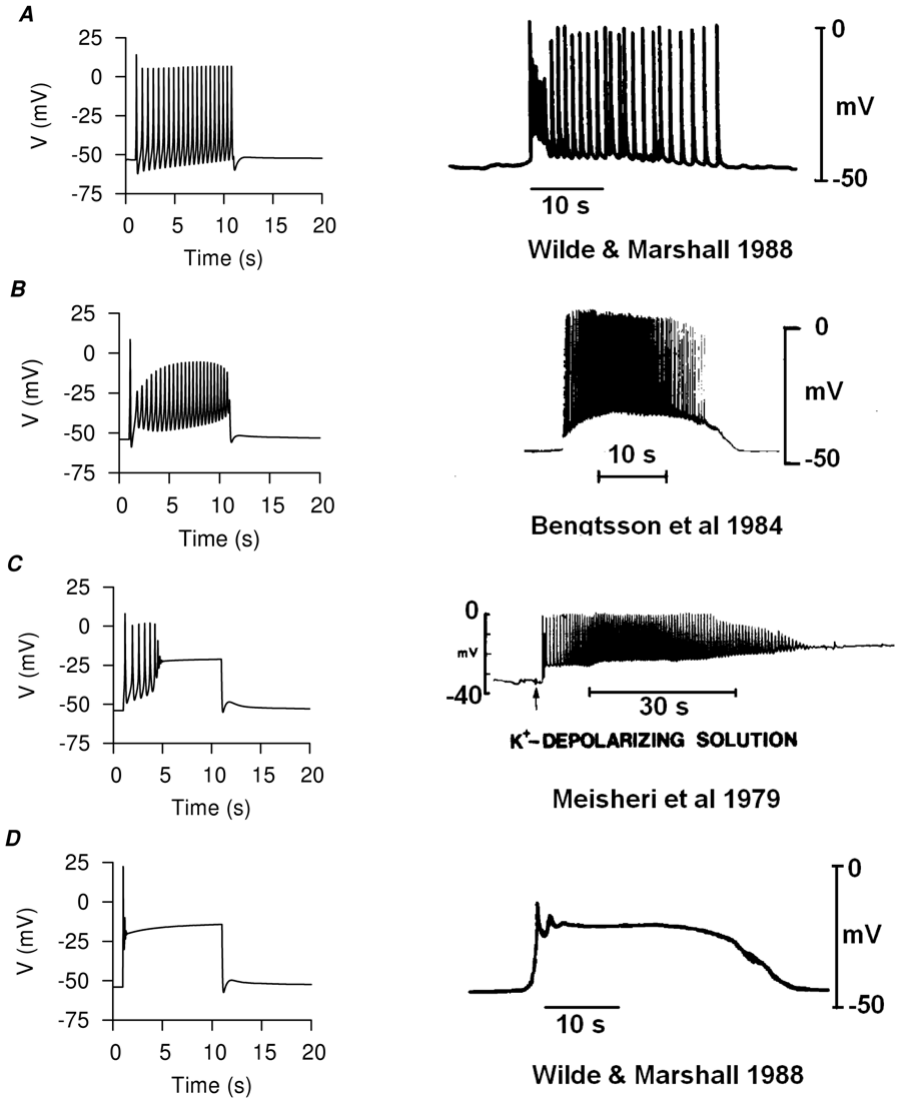
Varieties of action potentials. The USMC model can produce a range of myometrial action potentials (APs) using different initial conditions and parameters values. Four examples are shown (*left*); all four simulated APs were induced by a 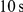 stimulus applied at 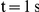. Representative experimental APs from published recordings [10], [101], [102] are shown for comparison (*right*). *A*, bursting type AP with afterpotentials at resting membrane potential (RMP); *B*, bursting type AP with depolarized afterpotentials; *C*, a mixed bursting-plateau type AP with initial repetitive spikes that gradually become a flat plateau at 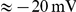. *D*, plateau type AP.

The range of AP shapes reported for the pregnant rat myometrium at 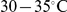 – repetitive spike AP [10], repetitive spike AP upon a depolarized basal membrane potential [101], repetitive spike AP leading to plateau [102] and a plateau-like AP [10] – are reconstructed in Figure 11. The variety of action potential shapes can be produced by this model with small variations in parameter sets and initial conditions. Of the four AP configurations illustrated in Figure 11: a bursting type AP was simulated with a current clamp of 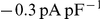 and with the conductance of 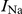 at 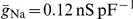; a bursting type AP upon a depolarized V was simulated with a current clamp of 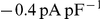 and with a slope factor of 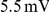 for the 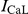 inactivation steady-state; a mixed bursting-plateau type AP was simulated with 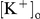 stepped from 6 to 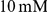; a plateau type AP was simulated with a current clamp of 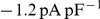. Thus the integrated model can accommodate a variety of APs seen in uterine in smooth muscle cells.

### Model validation 2: simulation of the experimental changes induced by estradiol on myometrial AP and 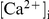 configurations

**Figure 12:**
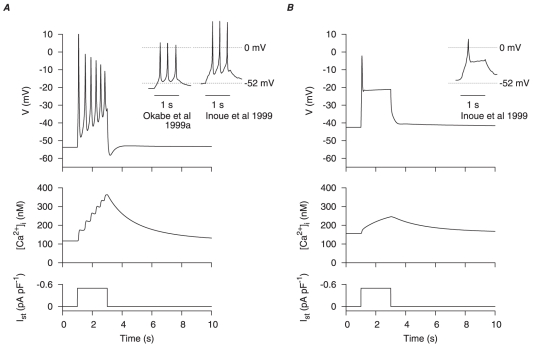
Simulating estradiol effects on simultaneous recordings of V and 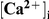. Action potentials (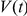) and corresponding calcium transients (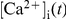) during a 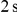 depolarizing current clamp (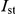) under, *A*, control conditions and, *B*, the effects of estradiol. In both cases, the initial conditions of the cell model were at their corresponding numerical equilibrium. Action potentials in rat longitudinal myometrial single cells under similar experimental conditions [11], [49] are shown for comparison (*insets*).

The cell model is validated with voltage-clamp and current-clamp experimental data from pregnant rat myometrial cells at 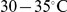 [11], [30], [32], [49] under control conditions and upon exposure to estradiol (Figure 12). Estradiol has been reported to reduce peak 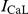. Estradiol has also been reported to reduce whole cell potassium currents [30], [32], [49] and change the USMC AP configuration from a bursting type AP upon a depolarized V to a plateau type AP [11]. The model was able to simulate this change in AP form by adjusting the appropriate current parameters: left-shifting the half-inactivation of 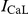 to 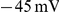 and alters its slope factor to 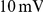, and reducing total potassium conductance by 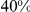 (Figure S1).

### Model validation 3: simulation of simultaneous recordings of membrane potential, 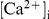 and force

*The extraction of the mathematical descriptions for modeling calcium-dependent force changes is denoted in Figure S6 and the resultant equations listed in the* *Methods* *(* *equations 3* *and* *8* *–* *9* *) and Appendix S1 (equations 104–105).*

**Figure 13:**
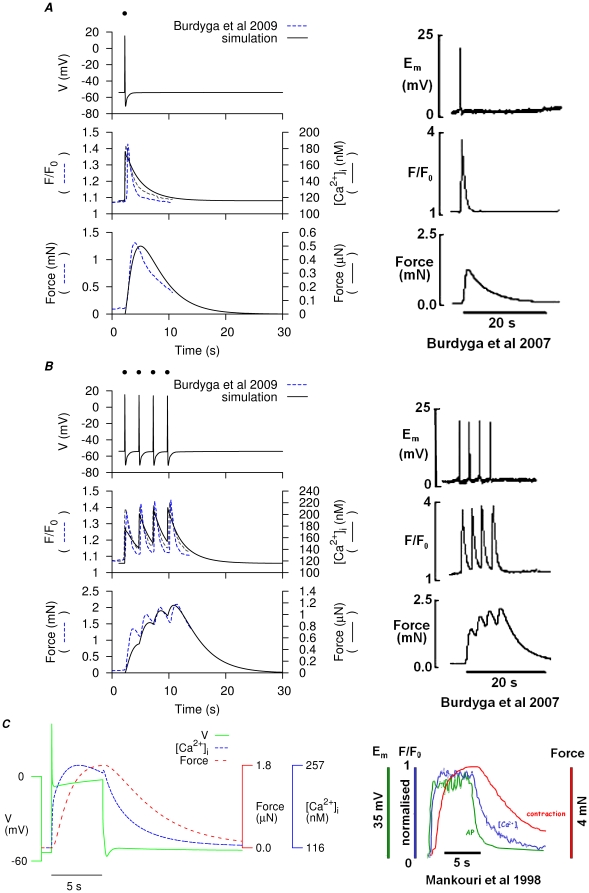
Simulation of the simultaneous recordings of myometrial V, 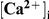 and force development. Simulated APs and corresponding 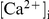 and force (*left*) compared to experimental simultaneous measurements of membrane potential, 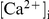 and force in rat myometrial tissue strips. *A*, simulation of a single spike AP and corresponding 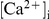 and force induced by a 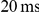 stimulus (*dot*) at 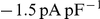 and compared to experimental data [103], [104]. *B*, four consecutive single spike APs and corresponding 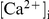 and force modeled by 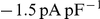 stimuli (*dots*) of 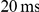, applied at 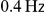 and compared to experimental data [103], [104]. *C*, superimposed simulated AP, 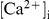 and force development (*left*), with a 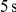 current clamp at 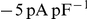 and compared to experimental data [106].

A final step in our validation of the model was to establish if it was able to accommodate the integration of uterine smooth muscle electrical, 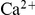 and contractile events necessary for excitation-contraction coupling. In this regard two broad scenarios of E-C coupling were again considered whereupon contractile events arose from either repetitive spike APs or from plateau-type APs. Figure 13 shows the results of simulations of APs, 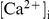 and force compared to published experimental measurements of these variables from rat myometrial tissue at 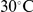 [103], [104]. Of note, we chose to reproduce the repetitive spike AP data with four separate consequent stimuli for two reasons. First, the present USMC model, when induced by a current clamp, exhibited a lower limit for bursting frequency at 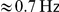 which was faster than that of the experimental recordings. Second, the experimental measurements of relative membrane potential changes from Burdyga *et al.*, [103], [104] were averaged from bundles of myometrial muscle strips. Thus, the low bursting frequency of spikes observed from these data may be a result of the extra electro-potential load from the multicellular environment. Alternatively, one cannot completely rule out the possibility that the four consecutive APs spikes were separate events resulting re-entrant excitation waves.

The model could also reproduce several additional published E-C coupling datasets of V(t), 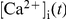 and force recorded from pregnant rats at 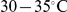 [103]–[106, ].Figures S7, S8

### Limitations and Conclusions

**Figure 14:**
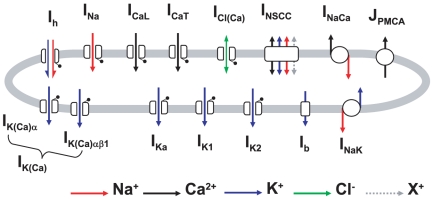
Schematic of the electrogenic components considered for the model of myometrial cell electrical excitability.

Our approach has resulted in a number of advances for our understanding of uterine smooth muscle E-C coupling. The model encompasses the most comprehensive biophysical description of ion channel and exchanger electric currents applied to the myometrium with 14 separate electrogenic components, summarized in Figure 14, used to simulate published myometrial AP forms and their alteration by specific experimental manoeuvres. Using 105 mathematical equations, it is the first model to integrate these electrogenic components with descriptions of 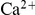 dynamics and phasic force production, the three essential components of electrical E-C coupling, and replicate published myometrial experimental recordings of simultaneous membrane potential, 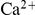 and force.

As with any mathematical model of biological phenomena there are limitations. The 14 electrogenic currents are likely to be an underestimate of the number of ion channel contributors to myometrial AP form. This highlights a lack of sufficient biophysical detail on other currents. In biophysical modeling of cells and tissues, it is often the case that some published electrophysiological information on particular currents is of insufficient detail to furnish biophysical modeling of all its steady-state and dynamic characteristics. Therefore, data from different resources with close cell types, or the same type of cells from different species, are used. This is the same case for the present model. The model is a hybrid containing information primarily from rat myometrium but also from human myometrium and cells expressing smooth muscle ion channel subunits, and this data has been obtained from experiments using different *in vitro* solutions and at different temperatures. Although this neglects any species-specific quantitative differences in uterine electrogenicity and E-C coupling, it presently is unavoidable. It is also common in biophysical modeling approaches when it is rare that all information is available for one cell type from one species. However, an advantage of the comprehensive assembly of this mathematical model is that it enables identification of gaps in our knowledge of myometrial electrogenesis. This will inform future empirical work in several ways.

First, the putative contribution of many ion channel subtypes to myometrial function has often been extrapolated from molecular data (mRNA or protein) which is incomplete (not all isotypes of channel or exchanger sub-families have been examined) or pharmacological data utilizing compounds of weak specificity (*e.g.* there are many putative pharmacological blockers/openers 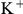 channel sub-family that have not yet been examined on uterine function). In addition, electrophysiological isolation of currents is often lacking. Clearly, identification of the molecular expression patterns of all ion channel and ion exchanger subtypes in myometrial cells of the uterus is essential (e.g. [107]), and marrying such data to precise electrophysiological, pharmacological and simulated profiles (even if initially this is in clonal cells), is required to furnish a complete biophysical characterization of normal uterine function. This should be accomplished for rodent and human myometrium to enable one to move from the present hybrid model to species-specific formulations. Procedures outlined in the development of this mathematical model indicate how this can assist in improving our understanding of uterine E-C coupling.

Second, from the present information, it is clear that isolated myometrial cells exhibit heterogeneity in ion channel electrophysiology and 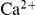 handling characteristics (for example, the proportion of examined cells exhibiting 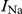 or particular 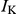 currents). It will be important as one moves forward to consider spatiotemporal aspects of E-C coupling that we establish the implications of this for tissue level electrogenesis [5], [108].

Third, the model serves as a useful tool in the design and assessment of agents that act as putative channel/exchanger blockers or activators. Refinement of the model with continued empirical/theoretical iterations will serve to increase its predictive capacity for use in the *in silico* assessment of new uterotonic agents especially as species-specific models are developed. For example, if electrophysiological data of sufficient detail for biophysical modeling is known for the actions of a new agonist/antagonist of a particular uterine ion channel then one can develop predictions of the likely action of this drug on uterine E-C coupling for that species. These will serve as hypotheses to be tested in *ex vivo* or *in vivo* experimentation. In the longer term, this should bring attendant benefits to developing drugs for the treatment of aberrant uterine activity such as preterm labor, whether experimentally induced in rodents [109] or arising spontaneously in humans, prolonged dysfunctional labor or poorly contracting uterus post-partum.

## Methods

### Overview

A mathematical model of uterine smooth muscle cell (USMC) function at late pregnancy was developed from the integration of data of individual ionic currents, calcium dynamics and contraction. A glossary of symbols used in the equations is given in Table S1. The USMC model is a system of first-order ordinary differential equations,
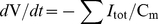
(1)

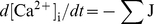
(2)

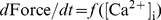
(3)where 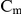 is the specific membrane capacitance. Eq. 1 describes the electrophysiological activities of myometrial membrane potential (V), which is proportional to the sum of membrane ionic currents (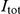); Eq. 2 describes the corresponding intracellular calcium (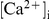) dynamics, which is proportional to the sum of calcium fluxes (J). Eq. 3 describes the rate of change of force as a function of 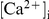.

### Electrophysiology

The individual membrane current components that were modeled were (i) four inward currents: L-type and T-type 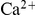 currents (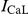, 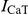), a fast inward 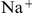 current (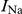) and a hyperpolarization-activated current (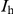); (ii) five outward currents: two voltage-gated 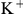 currents (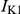, 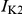), an A-type transient 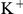 current (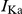) and two 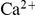-activated 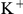 currents (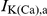, 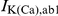); (iii) a non-specific cation current (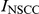); (iv) a 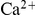-activated 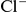 current (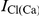); (v) a small background potassium current (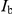); and (vi) an electrogenic 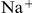-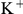 pump (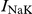) and a 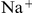-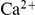 exchanger (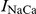). Properties of these currents are developed based on published voltage- and current-clamp experimental data of, wherever possible, late pregnant rat myometrial cells and tissues in the literature; where rat myometrial data is not available, but complementary data is available, *e.g.*, from human USMC, or clonal cells expressing rat-derived proteins, then this has been mentioned.

Most of the membrane currents were modeled with Hodgkin-Huxley type formulation in the following form:
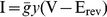
(4)

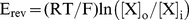
(5)

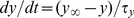
(6)where 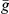 is maximum conductance, 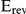 is the reversal potential, R is the universal gas constant, F is the Faraday constant, T is absolute temperature and 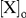 and 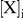 are the extracellular and intracellular ionic concentrations of ion X. The dimensionless gating variable (*y*) describes the time-dependent activation or inactivation profile of the channel conductance where 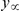, the steady-state value, and 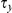, the time constant, are functions of voltage and/or ionic concentrations. For the electrogenic 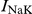 and 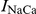, we adopted the formulations used in the description of cardiac ventricular cells from Nakao & Gadsby [87] and Weber *et al.*, [91] respectively. The nomenclature for the dynamic gating variables of individual membrane currents is listed in Table S2. Experimental data at body temperature, or a reported 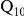 for an individual current, was available for 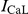, 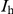, 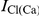, 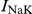 and 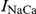. For other currents, we had to assume the simplest case whereby the dynamics were similar at both room and body temperature.

### Calcium dynamics

Bursztyn *et al.*, [24] modeled 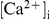 dynamics with three major calcium fluxes in myometrial cells: membrane calcium channels (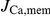), 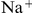-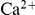 exchanger (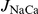) and plasma membrane 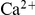 ATPase (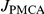) assuming 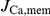 was at its equilibrium, *i.e.*, time-independent. Herein we have included the temporal dynamics of membrane calcium currents in 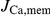, and adopted the Weber *et al.*, [91] formula for 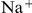-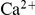 exchanger.


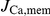
, which includes all the membrane calcium currents: 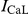, 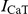 and the calcium component of 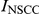 (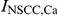), was calculated from the total membrane calcium current by
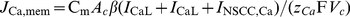
(7)where 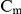 is the specific membrane capacitance; F is the Faraday constant; 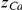 is the valency of 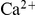 ions; 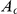 is the cell membrane surface area; 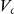 is cell volume; and 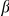 is the proportion of free intracellular 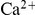 ions.

The geometry of a uterine smooth muscle cell is assumed to be two cone shapes joined end-to-end at their bases [110]. Reported cell sizes for late pregnant myometrial cells are 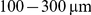 in length with a radius of 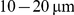 [29], [33]. As these dimensions cover a wide range, we represented the cell geometry with a single parameter, 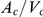, the surface area to volume ratio. We did not model cytoplasmic 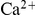 buffering proteins or intracellular calcium stores because such information for myometrial cells is too scant. Instead we assumed simply a tiny fraction of the membrane calcium influx to be free ions and the quantity is represented by the parameter 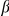 [111].

### Contractile mechanism

Force development during uterine contraction was modeled with a simple first-order ordinary differential equation:
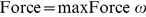
(8)

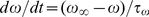
(9)where 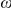 is the dimensionless gating variable describing the time-dependent activation profile of force, 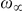 the steady-state value, and 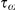 the time constant, are functions of 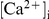. The force steady-state is described by the 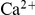-activated active force relationship from non-pregnant rat myometrium at 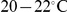 [112]; the time constant function is chosen to reproduce force development in late pregnant myometrial tissues recorded at 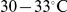 [103], [104] (Figure S6).

### Model simulations

Action potentials were induced in the whole cell model by applying an external stimulus current (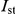), either as brief square pulses for single spike AP or with a current clamp for bursting or plateau AP.

The initial values of the dynamical variables (V, 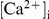, membrane current gating variables, and 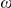) are listed in Table S3. The parameter values, which remain constant during simulations, are listed in Table S4. All the equations are given in Appendix S1.

Simulations were computed with a fixed time step of 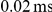, using XPPAUT [113] with either the fourth-order Runge-Kutta numerical integration method or the Euler Method, in a IBM laptop PC with a Intel(R) Pentium(R) M 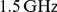 single processor. The Runge-Kutta was the method of choice for developing individual components and short simulations of the whole cell model whereas the Euler method was mainly used for simulations requiring longer integration times. Solutions of the whole USMC model using both integration methods are almost identical.

A copy of the model source code written in the C programming language is included in Appendix S2.

### Annotation of Figures

Within the body of some Figures there are textual annotations that mention the source references for the data plotted in those diagrams. Those references without parenthesis indicate published values that we have reproduced in the diagram. The references mentioned within parentheses reflect data that we have extracted from published raw tracings and refitted as displayed in the figures. Data referred to as ‘unpublished’ is remarked upon in the main text.

## Supporting Information

Figure S1Simulating the effect of estradiol on the inactivation of myometrial 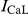.(PDF)

Figure S2Different inactivation kinetics of myometrial 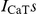.(PDF)

Figure S3Divalent ion concentration versus 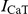 inactivation time constant of rat myometrial 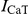.(PDF)

Figure S4Experimental current tracings of 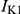 from five cells and an example of extrapolated 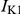 at the voltage step (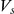) 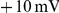 averaged from these five cells.(PDF)

Figure S5Experimental current tracings of 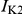 from four cells and an example of extrapolated 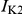 at the voltage step (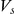) 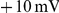 averaged from these four cells.(PDF)

Figure S6Modeling the dynamics of 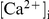-dependent active force.(PDF)

Figure S7Modeling simultaneous changes in V and 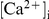 or 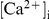 and force development.(PDF)

Figure S8 Modeling force output consequent to spike APs or plateau-like APs.
(PDF)

Table S1 Definitions of the equation symbols.
(PDF)

Table S2 Definition of gating variables for individual currents and force, and the corresponding experimental temperature and species.
(PDF)

Table S3 Initial values of the dynamics variables used in model simulations.
(PDF)

Table S4 Constant parameter values used in model simulations.
(PDF)

Appendix S1 Equations used in the model simulations.
(PDF)

Appendix S2 Model source code.
(BZ2)
