# Correction: A Computational Model of the Ionic Currents, Ca^2+^ Dynamics and Action Potentials Underlying Contraction of Isolated Uterine Smooth Muscle

**DOI:** 10.1371/annotation/d317e049-4927-4906-95a5-cd0198a3feb9

**Published:** 2011-10-07

**Authors:** Wing-Chiu Tong, Cecilia Y. Choi, Sanjay Kharche, Arun V. Holden, Henggui Zhang, Michael J. Taggart

The correct spelling of the 3rd author's name is: Sanjay Kharche. The correct citation is: Tong W-C, Choi CY, Kharche S, Holden AV, Zhang H, et al. (2011) A Computational Model of the Ionic Currents, Ca2+ Dynamics and Action Potentials Underlying Contraction of Isolated Uterine Smooth Muscle. PLoS ONE 6(4): e18685. doi:10.1371/journal.pone.0018685. 

