# Supplementary material for: A Computational Model of the Ionic Currents, Ca2+ Dynamics and Action Potentials Underlying Contraction of Isolated Uterine Smooth Muscle
Source: PLoS One. 2011 Apr 29;6(4):e18685. doi: 10.1371/journal.pone.0018685 (PMC3084699; doi:10.1371/journal.pone.0018685)
Supplement: Figure S1 — Simulating the effect of estradiol on the inactivation of myometrial . (PDF) [file pone.0018685.s001.pdf]

Supporting Information

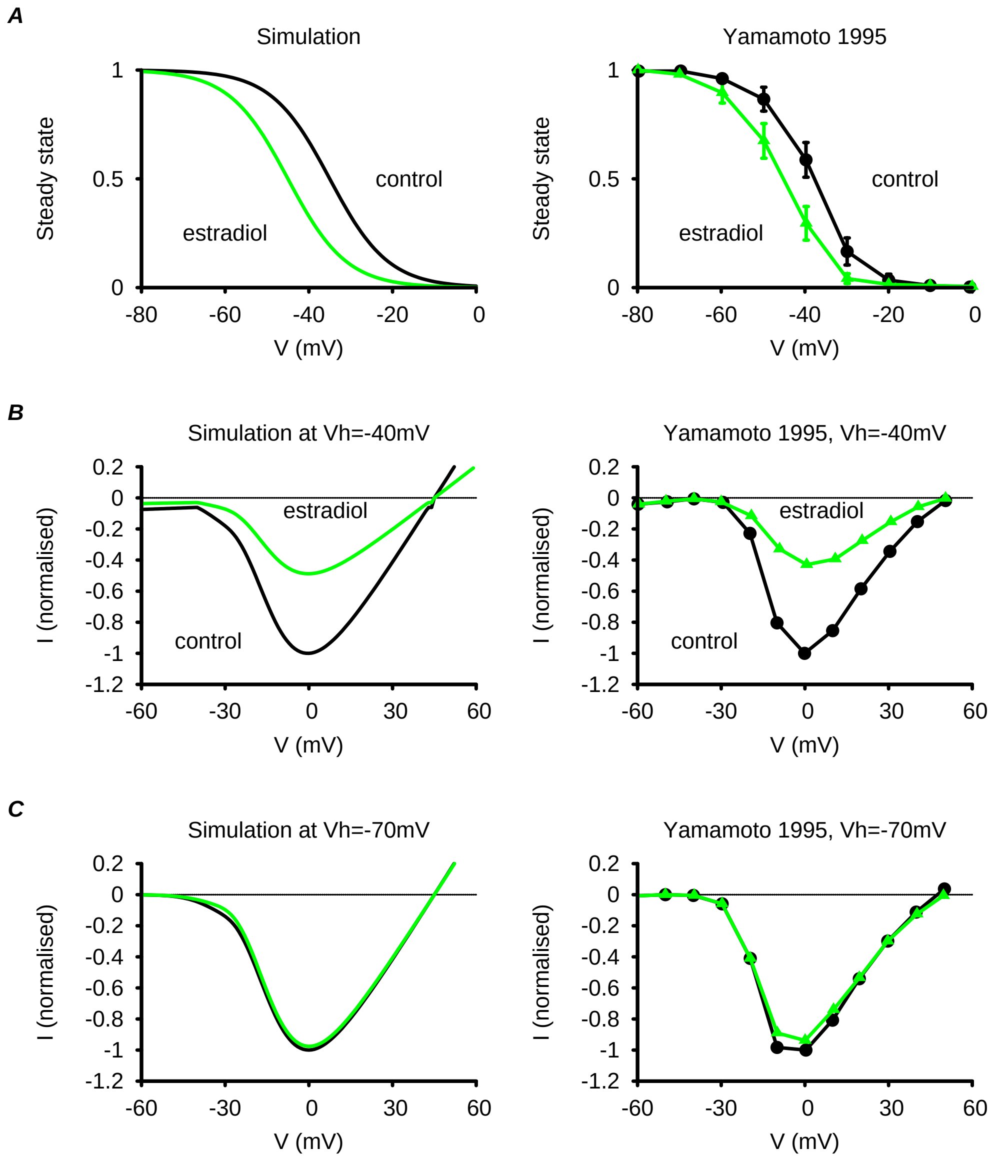

Figure S1. Simulating the effect of estradiol on the inactivation of myometrial I_{CaL}. Simulations (left panels) reproduced the experimental data (right panels, [30]) indicating that estradiol effects a leftward shift in voltage-dependent inactivation and a reduction in peak current-voltage (I-V) relationship from a holding potential (V_h) of -40 mV. I_{CaL} in control conditions (*black*) and I_{CaL} in estradiol conditions (*green*). *A*, inactivation steady-states of I_{CaL}; *B*, I-V relationship from $V_h = -40$ mV; *C*, I-V relationship from $V_h = -70$ mV.
