# Supplementary material for: A Computational Model of the Ionic Currents, Ca2+ Dynamics and Action Potentials Underlying Contraction of Isolated Uterine Smooth Muscle
Source: PLoS One. 2011 Apr 29;6(4):e18685. doi: 10.1371/journal.pone.0018685 (PMC3084699; doi:10.1371/journal.pone.0018685)
Supplement: Figure S2 — Different inactivation kinetics of myometrial . (PDF) [file pone.0018685.s002.pdf]

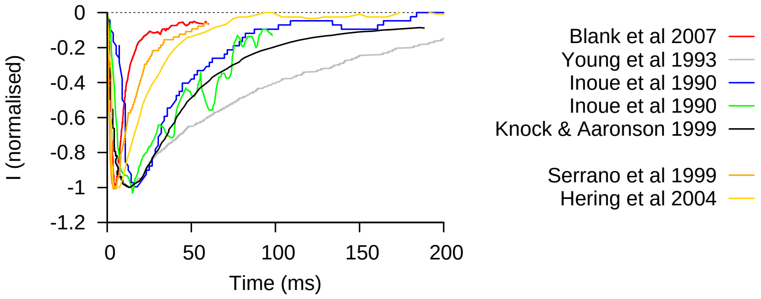

References	Cell types	ion	T (°C)	V_h (mV)	V_s (mV)	Identification Methods
Blank *et al* 2007	human myometrial cells	Ca^{2+}	22	-80	-30	Nickel, and difference between $V_h = -80$ mV and $V_h = -50$ mV
Young *et al* 1993	human myometrial cells	Ca^{2+}	22	-80	-20	
Inoue *et al* 1990	human myometrial cells	Ca^{2+}	RT	-100	-20, -40	difference between $V_h = -100$ mV and $V_h = -50$ mV
Knock & Aaronson 1999	human myometrial cells	Ba^{2+}	22	-80	-20	Mibefradil
Serrano *et al* 1999	Rat α1G gene in HEK cells	Ca^{2+}	20	-100	-30	
Hering *et al* 2004	Rat Cav3.1 gene in HEK cells	Ca^{2+}	RT	-100	-20	

Figure S2. Different inactivation kinetics of myometrial I_{CaT}. Experimental time tracings of myometrial ion currents described as I_{CaT} from human [13, 18, 28, 47] and rat [43, 44] showed a broad range of inactivation kinetics. All tracings are normalized to their peak value. The table compares specific experimental conditions for each I_{CaT} description: the cell types, carrier ion, temperature (T), holding potential (V_h), stepping potential (V_s), and methods of I_{CaT} identification.
