# Supplementary material for: A Computational Model of the Ionic Currents, Ca2+ Dynamics and Action Potentials Underlying Contraction of Isolated Uterine Smooth Muscle
Source: PLoS One. 2011 Apr 29;6(4):e18685. doi: 10.1371/journal.pone.0018685 (PMC3084699; doi:10.1371/journal.pone.0018685)
Supplement: Figure S3 — Divalent ion concentration versus inactivation time constant of rat myometrial . (PDF) [file pone.0018685.s003.pdf]

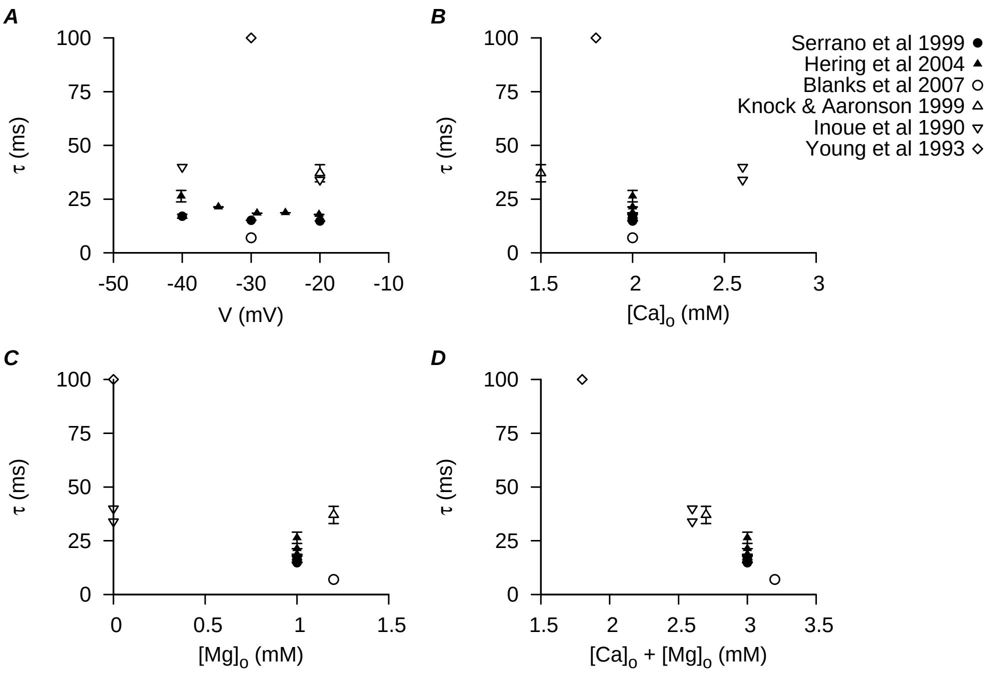

References	V_h (mV)	V_s (mV)	τ (ms)	$[Ca^{2+}]_o$ (mM)	$[Mg^{2+}]_o$ (mM)
Blank *et al* 2007	-80	-30	7	2	1.2
Young *et al* 1993	-100	-30	100	1.8	0
Inoue *et al* 1990	-100	-20	34	2.6	0
Inoue *et al* 1990	-100	-40	40	2.6	0
Knock & Aaronson 1999	-80	-20	37 ± 4	1.5	1.2
Serrano *et al* 1999	-100	-40	17.03 ± 0.93	2	1
Serrano *et al* 1999	-100	-30	15.17	2	1
Serrano *et al* 1999	-100	-20	14.77	2	1
Hering *et al* 2004	-100	-40	26.37 ± 2.64	2	1
Hering *et al* 2004	-100	-35	21.32	2	1
Hering *et al* 2004	-100	-30	18.46	2	1
Hering *et al* 2004	-100	-25	18.68	2	1
Hering *et al* 2004	-100	-20	17.80	2	1

Figure S3. Divalent ion concentration versus I_{CaT} inactivation time constant of rat myometrial I_{CaT}. The voltage-dependent time constants of inactivation (τ) for myometrial I_{CaT} from published records were compared against the voltage step conditions and divalent ion concentrations ($[Mg^{2+}]_o$ and $[Ca^{2+}]_o$) used in each of the experimental scenarios. τ is either taken from the published values or by fitting published raw data tracings with an exponential function from [13, 18, 28, 43, 44, 47]. *A*, τ with respect to the stepping voltage V_s from a holding potential V_h of either -80 mV or -100 mV. *B*, τ with respect to $[Ca^{2+}]_o$; *C*, τ with respect to $[Mg^{2+}]_o$; *D*, τ with respect to sum of $[Ca^{2+}]_o$ and $[Mg^{2+}]_o$. The inactivation time constant of I_{CaT} show a stronger association with the combined concentrations of $[Ca^{2+}]_o$ and $[Mg^{2+}]_o$ than with only $[Mg^{2+}]_o$.
