# Supplementary material for: A Computational Model of the Ionic Currents, Ca2+ Dynamics and Action Potentials Underlying Contraction of Isolated Uterine Smooth Muscle
Source: PLoS One. 2011 Apr 29;6(4):e18685. doi: 10.1371/journal.pone.0018685 (PMC3084699; doi:10.1371/journal.pone.0018685)
Supplement: Figure S4 — Experimental current tracings of from five cells and an example of extrapolated at the voltage step () averaged from these five cells. (PDF) [file pone.0018685.s004.pdf]

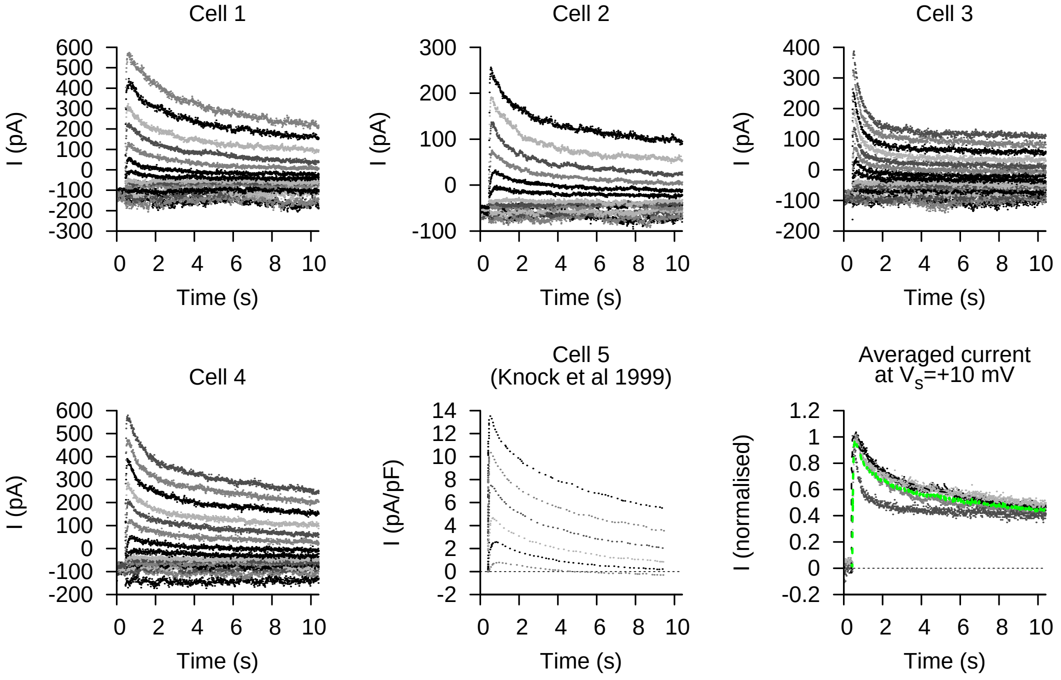


**Figure S4. Experimental current tracings of $I_{\mathrm{K1}}$ from five cells and an example of extrapolated $I_{\mathrm{K1}}$ at the voltage step ($V_s$) +10 mV averaged from these five cells.** The data for *Cell 1-4* are unpublished data provided by Knock G & Aaronson P (personal communication); data for the fifth cell is from Knock *et al.*, [19]. $I_{\mathrm{K1}}$ currents were induced in these cells during a 10 s voltage-clamp at different $V_s$ from a holding potential ($V_h$) of −80 mV. The membrane potentials were increment every 10 mV steps from −100 mV to: +40 mV (*Cell 1*), +30 mV (*Cell 2*), +50 mV (*Cell 3, Cell 4*), and from −40 mV to +10 mV in Knock *et al.*, [19]; each $V_s$ is separated by a different shade of gray. The steps taken to produce the averaged current tracings for $I_{\mathrm{K1}}$ from −40 mV to +30 mV were as follows. For each current tracing in *Cell 1-5*, the values are first offset to zero (so that the current tracing is at zero during $V_h$), then normalized to the peak value at $V_s$= +10 mV. At each $V_s$, the current values at each time instant from different cells were summed and divided by the total number of cells (for −40 mV to +10 mV, n=5; for +20 mV to +30 mV, n=4); this gave an averaged current tracing for a specific $V_s$. The example shows an extrapolated $I_{\mathrm{K1}}$ at $V_s$= +10 mV (*green*), superimposed with the tracings from the five cells at the same $V_s$ (*different shade of gray*). The averaged current tracings were used for extracting activation and inactivation time constants and for validation of $I_{\mathrm{K1}}$ under voltage-clamp conditions.
