# Supplementary material for: A Computational Model of the Ionic Currents, Ca2+ Dynamics and Action Potentials Underlying Contraction of Isolated Uterine Smooth Muscle
Source: PLoS One. 2011 Apr 29;6(4):e18685. doi: 10.1371/journal.pone.0018685 (PMC3084699; doi:10.1371/journal.pone.0018685)
Supplement: Figure S5 — Experimental current tracings of from four cells and an example of extrapolated at the voltage step () averaged from these four cells. (PDF) [file pone.0018685.s005.pdf]

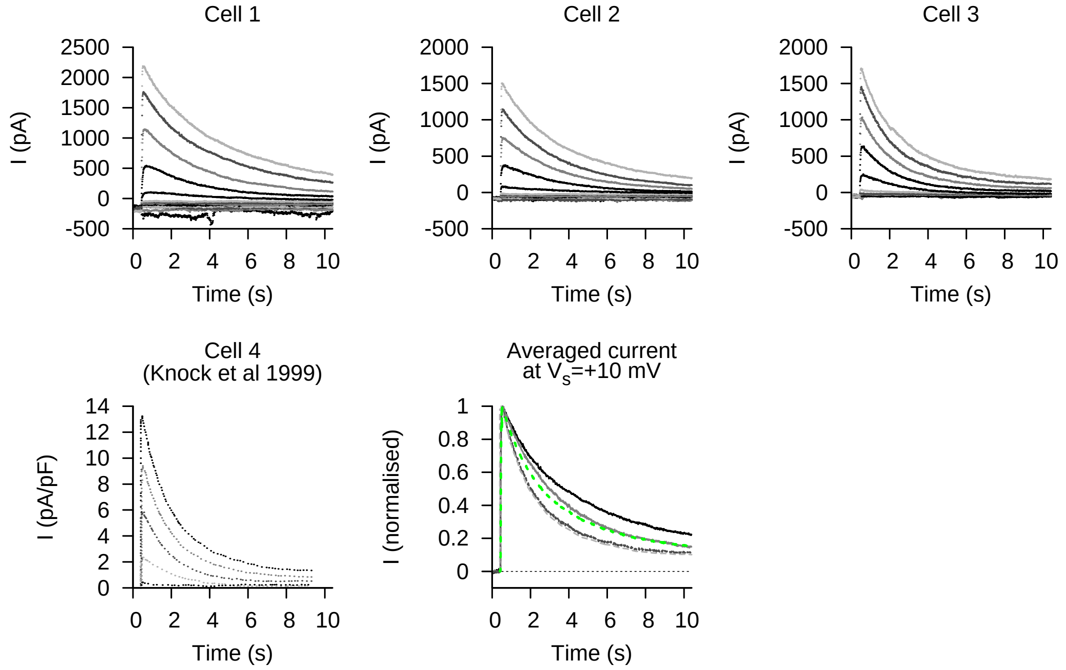

Figure S5. Experimental current tracings of I_{K2} from four cells and an example of extrapolated I_{K2} at the voltage step (V_s) $+10\,\mathrm{mV}$ averaged from these four cells. The data for *Cell 1-3* are unpublished data provided by Knock G & Aaronson P (personal communication); data for the fourth cell is from Knock *et al.*, [19]. I_{K2} were induced in these cells during a 10 s voltage-clamp at different V_s from a holding potential (V_h) of $-80\,\mathrm{mV}$. The membrane potentials were increment every 10 mV steps from $-100\,\mathrm{mV}$ to $+20\,\mathrm{mV}$ (*Cell 1-3*) and from $-40\,\mathrm{mV}$ to $+10\,\mathrm{mV}$ in Knock *et al.*, [19]; each V_s is separated by a different shade of gray. The steps taken to produce the averaged current tracings for I_{K2} from $-40\,\mathrm{mV}$ to $+20\,\mathrm{mV}$ were as follows. For each current tracing in *Cell 1-3*, the values are first offset to zero (so that the current tracing is at zero during V_h), then normalized to the peak value at $V_s = +10\,\mathrm{mV}$. At each V_s, the current values at each time instant from different cells were summed and divided by the total number of cells (for $-40\,\mathrm{mV}$ to $+20\,\mathrm{mV}$, n=4); this gave an averaged current tracing for a specific V_s. The example shows an extrapolated I_{K2} at $V_s = +10\,\mathrm{mV}$ (*green*), superimposed with the tracings from the four cells at the same V_s (*different shade of gray*). The averaged current tracings were used for extracting activation and inactivation time constants and for validation of I_{K2} under voltage-clamp conditions.
