# Supplementary material for: A Computational Model of the Ionic Currents, Ca2+ Dynamics and Action Potentials Underlying Contraction of Isolated Uterine Smooth Muscle
Source: PLoS One. 2011 Apr 29;6(4):e18685. doi: 10.1371/journal.pone.0018685 (PMC3084699; doi:10.1371/journal.pone.0018685)
Supplement: Figure S6 — Modeling the dynamics of -dependent active force. (PDF) [file pone.0018685.s006.pdf]

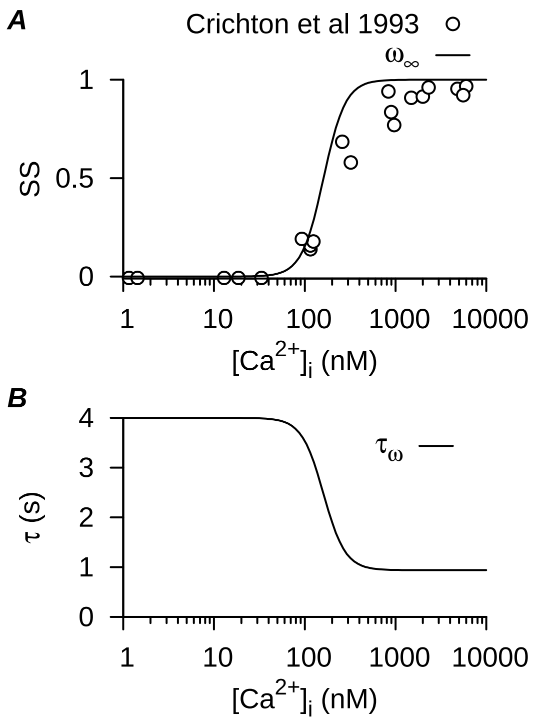

Figure S6. Modeling the dynamics of $[Ca^{2+}]_i$**-dependent active force.** $[Ca^{2+}]_i$-dependent active force is modeled by a first-ordered differential equation with a dynamical variable (ω) with both its steady-state (ω_∞) and time constant (τ_ω) dependent on $[Ca^{2+}]_i$. *A*, ω_∞ was first approximated using results from Crichton *et al.*, [115] then adjusted to best reproduce results from Burdyga *et al.*, [106, 107]; it is fitted using a Hill equation with a half-activation at 161.3 nM and a Hill coefficient of 3.6 (Eq. 104). *B*, the equation for τ_w (Eq. 105) was chosen to best reproduce experimental results [106, 107] and gave a maximum of 4 s at low $[Ca^{2+}]_i$ and a minimum of 0.94 s at high $[Ca^{2+}]_i$.
