# Supplementary material for: A Computational Model of the Ionic Currents, Ca2+ Dynamics and Action Potentials Underlying Contraction of Isolated Uterine Smooth Muscle
Source: PLoS One. 2011 Apr 29;6(4):e18685. doi: 10.1371/journal.pone.0018685 (PMC3084699; doi:10.1371/journal.pone.0018685)
Supplement: Figure S7 — Modeling simultaneous changes in V and or and force development. (PDF) [file pone.0018685.s007.pdf]

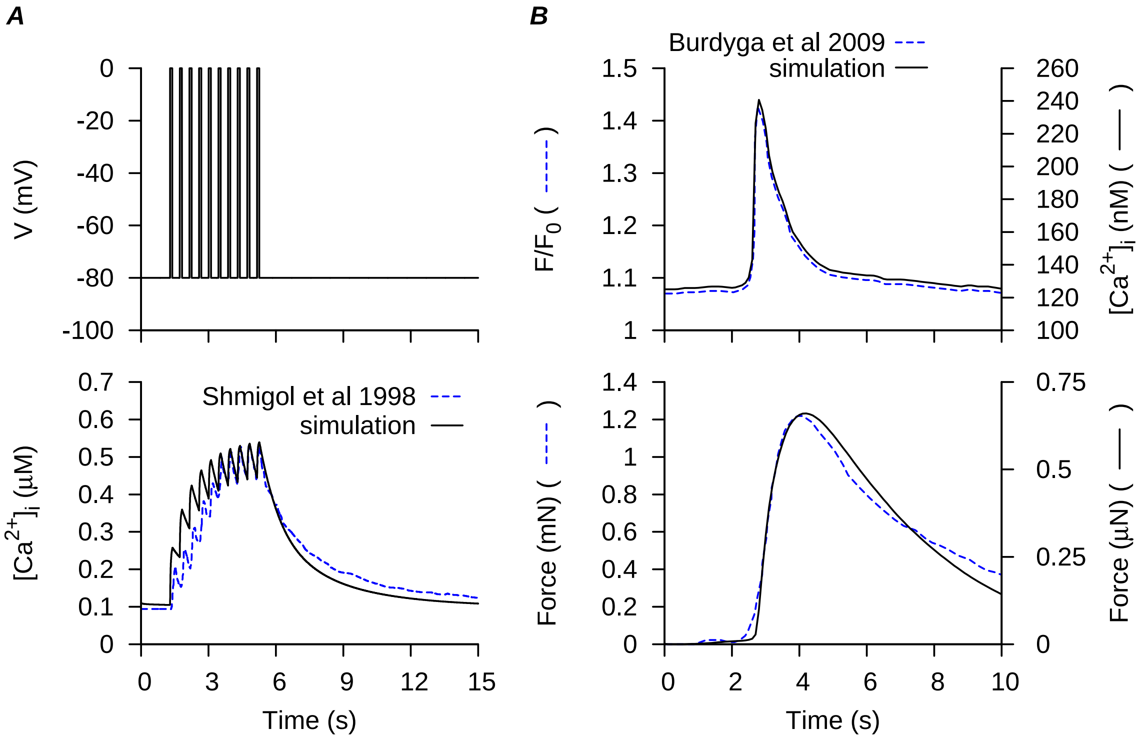

Figure S7. Modeling simultaneous changes in V and $[Ca^{2+}]_i$ or $[Ca^{2+}]_i$ and force development. *A*, the modified calcium sub-system was subjected to a burst of ten voltage-clamp pulses from a holding potential of -80 mV to 0 mV for 100 ms at 3 Hz (*top panel*); the evoked calcium tracings (*bottom panel, solid line*) exhibited similar dynamics as experimental results in Shmigol *et al.*, [12] (*dash line*). *B*, the contraction component was commanded by the experimental calcium tracing as control input (*top panel*); values of calcium measurement were transformed into concentration values by assuming $[Ca^{2+}]_i = 125$ nM at rest and ≈ 240 nM at peak when evoked by a single AP. The evoked force tracings (*solid line*) exhibited similar temporal dynamics as experimental results in tissue (*dash line*) (*bottom panel*) [107]; the resting force in the single cell model is set as zero at rest.
