# Supplementary material for: A Computational Model of the Ionic Currents, Ca2+ Dynamics and Action Potentials Underlying Contraction of Isolated Uterine Smooth Muscle
Source: PLoS One. 2011 Apr 29;6(4):e18685. doi: 10.1371/journal.pone.0018685 (PMC3084699; doi:10.1371/journal.pone.0018685)
Supplement: Figure S8 — Modeling force output consequent to spike APs or plateau-like APs. (PDF) [file pone.0018685.s008.pdf]

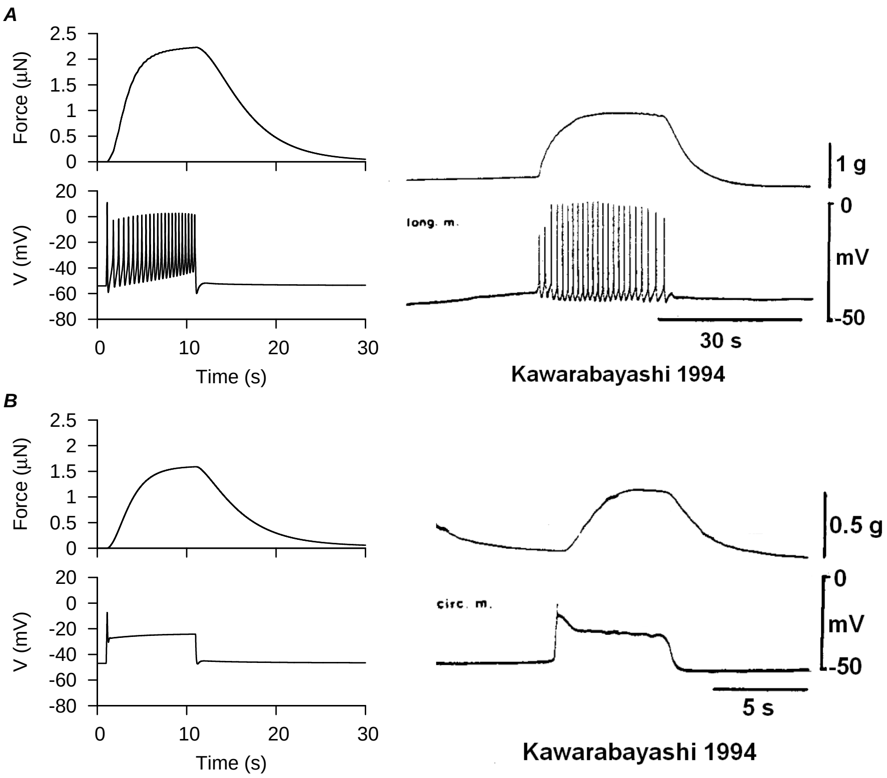

Figure S8. Modeling force output consequent to spike APs or plateau-like APs. *A*, a simulated bursting AP and consequent force induced with a 10 s current clamp at $-0.4\,\mathrm{pA\,pF^{-1}}$ in comparison to experimental data of rat longitudinal muscle (*right*) [108]. *B*, a simulated plateau-type AP and consequent force (*left*) instigated with a 10 s current clamp at $-0.4\,\mathrm{pA\,pF^{-1}}$, reduced potassium conductances ($\bar{g}_{\mathrm{K}}=\bar{g}_{\mathrm{KCa}}= 0.55\,\mathrm{nS\,pF^{-1}}$) and left-shifted I_{CaL} half inactivation at $-50\,\mathrm{mV}$ compared to panel A, in comparison to experimental data(*right*) [108].
