# Supplementary material for: A Computational Model of the Ionic Currents, Ca2+ Dynamics and Action Potentials Underlying Contraction of Isolated Uterine Smooth Muscle
Source: PLoS One. 2011 Apr 29;6(4):e18685. doi: 10.1371/journal.pone.0018685 (PMC3084699; doi:10.1371/journal.pone.0018685)
Supplement: Table S1 — Definitions of the equation symbols. (PDF) [file pone.0018685.s009.pdf]

Table S1. Definitions of the equation symbols

Symbols	meaning
V	membrane potential
$V_{0.5}$	half (in)activation constant
V_h	holding potential
V_s	voltage steps
A_c	cell surface area
V_c	cell volume
C_m	specific membrane capacitance
R	the universal gas constant
F	the Faraday constant
T	absolute temperature
Q_{10}	Temperature coefficient
ΔT	Temperature change
Ca^{2+}	calcium ion
Na^+	sodium ion
K^+	potassium ion
Cl^-	chloride ion
Mg^{2+}	magnesium ion
Cs^+	cesium ion
NMGD	N-methyl-D-glucamine
z_{Na}	Na^+ valency
z_{Ca}	Ca^{2+} valency
z_K	K^+ valency
β	Proportion of free Ca^{2+} ions
[X]i	intracellular concentration of ion X
[X]o	extracellular concentration of ion X
$[Ca^{2+}]_\mathrm{i}$	intracellular calcium
$[Ca^{2+}]_\mathrm{o}$	extracellular calcium
$[Na^+]_\mathrm{i}$	intracellular sodium
$[Na^+]_\mathrm{o}$	extracellular sodium
$[K^+]_\mathrm{i}$	intracellular potassium
$[K^+]_\mathrm{o}$	extracellular potassium
$[Cl^-]_\mathrm{i}$	intracellular chloride
$[Cl^-]_\mathrm{o}$	extracellular chloride
$[Mg^{2+}]_\mathrm{o}$	extracellular magnesium
$[Cs^+]_\mathrm{i}$	intracellular cesium
$[Cs^+]_\mathrm{o}$	extracellular cesium
[NMGD]	concentration of NMGD
E_rev	reversal potential
E_CaL	reversal potential of I_CaL
E_CaT	reversal potential of I_CaT
E_Na	reversal potential of I_Na
E_h	reversal potential of I_h
E_K	reversal potential of potassium currents
E_Cl	reversal potential of $I_\mathrm{Cl(Ca)}$
E_NS	reversal potential of I_NSCC

Continued on next page

Table S1 – continued from previous page

Symbols	meaning
P_{Ca}	Permeability of calcium, ratio of flux concentration with respect to cesium
P'_{Ca}	Permeability of calcium, corrected with the effect of the electric field
P_{Na}	Permeability of sodium
P_{K}	Permeability of potassium
P_{Cs}	Permeability of cesium
P_{NMGD}	Permeability of NMGD
I	current
I_{ion}	total membrane ionic currents
I_{CaL}	L-type Ca^{2+} current
I_{CaT}	T-type Ca^{2+} current
I_{Na}	fast inward Na^{+} current
I_{h}	hyperpolarization-activated current
I_{K1}	voltage-gated K^{+} current, inactivate with $V_h \geq -40\,\mathrm{mV}$
I_{K2}	voltage-gated K^{+} current, inactivate with $V_h \geq 0\,\mathrm{mV}$
I_{Ka}	A-type transient K^{+} current
$I_{\mathrm{K(Ca)}}$	total Ca^{2+}-activated K^{+} current
I_{α}	$I_{\mathrm{K(Ca)}}$ subtype, consists of only α subunits
$I_{\alpha\beta1}$	$I_{\mathrm{K(Ca)}}$ subtype, consists of α and $\beta 1$ subunits
I_{b}	background K^{+} current
$I_{\mathrm{Cl(Ca)}}$	Ca^{2+}-activated Cl^{-} currents
I_{NSCC}	non-specific cation current
$I_{\mathrm{NSCC,Ca}}$	calcium component of I_{NSCC}
I_{NaK}	Na^{+}-K^{+} pump current
I_{NaCa}	Na^{+}-Ca^{2+} exchanger current
J	Ca^{2+} flux
$J_{\mathrm{Ca,mem}}$	Ca^{2+} flux *via* membrane Ca^{2+} currents
J_{PMCA}	Ca^{2+} flux *via* plasma membrane Ca^{2+}-ATPase
J_{NaCa}	Ca^{2+} flux *via* Na^{+}-Ca^{2+} exchanger
$\bar{J}_{\mathrm{PMCA}}$	maximal J_{PMCA}
$\bar{J}_{\mathrm{NaCa}}$	maximal J_{NaCa}
$\bar{g}$	maximum conductance
$\bar{g}_{\mathrm{CaL}}$	maximum conductance of I_{CaL}
$\bar{g}_{\mathrm{CaT}}$	maximum conductance of I_{CaT}
$\bar{g}_{\mathrm{Na}}$	maximum conductance of I_{Na}
$\bar{g}_{\mathrm{h}}$	maximum conductance of I_{h}
$\bar{g}_{\mathrm{K1}}$	maximum conductance of I_{K1}
$\bar{g}_{\mathrm{K2}}$	maximum conductance of I_{K2}
$\bar{g}_{\mathrm{Ka}}$	maximum conductance of I_{Ka}
$\bar{g}_{\mathrm{K(Ca)}}$	maximum conductance of $I_{\mathrm{K(Ca)}}$
$\bar{g}_{\mathrm{b}}$	maximum conductance of I_{b}
$\bar{g}_{\mathrm{Cl(Ca)}}$	maximum conductance of $I_{\mathrm{Cl(Ca)}}$
$\bar{g}_{\mathrm{NS}}$	maximum conductance of I_{NSCC}
$\bar{g}_{\mathrm{L}}$	maximum conductance of the leak component in I_{NSCC}
$\bar{g}_{\mathrm{NaK}}$	maximum conductance of I_{NaK}
g_{NS}	conductance of I_{NSCC}

Continued on next page

Table S1 – continued from previous page

Symbols	meaning
g_s	numerical constant, used for normalizing dependency of extracellular ions on the conductance of I_{NSCC}
n_{K}	Hill coefficient of $[\mathrm{K}^+]_{\mathrm{o}}$ dependency of I_{NaK}
n_{Na}	Hill coefficient of $[\mathrm{Na}^+]_{\mathrm{i}}$ dependency of I_{NaK}
n_{PMCA}	Hill coefficient of plasma membrane Ca^{2+}-ATPase
n_{Allo}	Hill coefficient of allosteric regulation of Na^+-Ca^{2+} exchanger
n_{F}	Hill coefficient of the steady-state of calcium-dependent active force
f_{Ca}	Calcium inhibition of I_{CaL}
f_{Mg}	Magnesium inhibition of I_{NSCC}
f_{NaK}	Voltage-dependency of I_{NaK}
n_{NaK}	$[\mathrm{Na}^+]_{\mathrm{i}}$ dependency of I_{NaK}
k_{NaK}	$[\mathrm{K}^+]_{\mathrm{o}}$ dependency of I_{NaK}
k_{sat}	Saturation factor for Na^+-Ca^{2+} exchanger at very negative potential
$f_{1,NaCa}$	Potential dependency of J_{NaCa}
$f_{2,NaCa}$	Potential dependency of J_{NaCa}
f_{allo}	Allosteric regulation by $[\mathrm{Ca}^{2+}]_{\mathrm{i}}$ of Na^+-Ca^{2+} exchanger
γ	Partition parameter of J_{NaCa}
p_a	proportion of I_α in $I_{\mathrm{K(Ca)}}$
p_b	proportion of $I_{\alpha\beta1}$ in $I_{\mathrm{K(Ca)}}$
$\mathrm{K}_{1,\mathrm{Cl}}$	Equilibrium constant for the calcium-binding property of c_∞ of $I_{\mathrm{Cl(Ca)}}$
$\mathrm{K}_{2,\mathrm{Cl}}$	Equilibrium constant for the voltage-dependency of c_∞ of $I_{\mathrm{Cl(Ca)}}$
$\mathrm{K}_{d,\mathrm{CaL}}$	Half-saturation concentration for $[\mathrm{Ca}^{2+}]_{\mathrm{i}}$ inhibition of I_{CaL}
$\mathrm{K}_{d,\mathrm{Mg}}$	Half-saturation concentration for $[\mathrm{Mg}^{2+}]_{\mathrm{o}}$ inhibition of I_{NSCC}
$\mathrm{K}_{m,\mathrm{K}}$	Half-saturation concentration for $[\mathrm{K}^+]_{\mathrm{o}}$ dependency of I_{NaK}
$\mathrm{K}_{m,\mathrm{Na}}$	Half-saturation concentration for $[\mathrm{Na}^+]_{\mathrm{i}}$ dependency of I_{NaK}
$\mathrm{K}_{m,\mathrm{PMCA}}$	Half-saturation concentration for $[\mathrm{Ca}^{2+}]_{\mathrm{i}}$ association of plasma membrane Ca^{2+}-ATPase
$\mathrm{K}_{m,\mathrm{Allo}}$	Half-saturation concentration for $[\mathrm{Ca}^{2+}]_{\mathrm{i}}$ allosteric factor of Na^+-Ca^{2+} exchanger
$\mathrm{K}_{m,\mathrm{Cai}}$	Dissociation constant for $[\mathrm{Ca}^{2+}]_{\mathrm{i}}$ of Na^+-Ca^{2+} exchanger
$\mathrm{K}_{m,\mathrm{Cao}}$	Dissociation constant for $[\mathrm{Ca}^{2+}]_{\mathrm{o}}$ of Na^+-Ca^{2+} exchanger
$\mathrm{K}_{m,\mathrm{Nai}}$	Dissociation constant for $[\mathrm{Na}^+]_{\mathrm{i}}$ of Na^+-Ca^{2+} exchanger
$\mathrm{K}_{m,\mathrm{Nao}}$	Dissociation constant for $[\mathrm{Na}^+]_{\mathrm{o}}$ of Na^+-Ca^{2+} exchanger
$\mathrm{K}_{m,\mathrm{F}}$	Apparent affinity constant for $[\mathrm{Ca}^{2+}]_{\mathrm{i}}$ of contraction
b	Activation gate of I_{CaT}
c	Activation gate of $I_{\mathrm{Cl(Ca)}}$
d	Activation gate of I_{CaL}
f_1	Fast inactivation gate of I_{CaL}
f_2	Slow inactivation gate of I_{CaL}
g	Inactivation gate of I_{CaT}
h	Inactivation gate of I_{Na}
m	Activation gate of I_{Na}
p	Activation gate of I_{K2}
k_1	Fast inactivation gate of I_{K2}
k_2	Slow inactivation gate of I_{K2}
q	Activation gate of I_{K1}

Continued on next page

Table S1 – continued from previous page

Symbols	meaning
r_1	Fast inactivation gate of I_{K1}
r_2	Slow inactivation gate of I_{K1}
s	Activation gate of I_{Ka}
x	Inactivation gate of I_{Ka}
$x_{\alpha\beta1}$	Activation gate of $I_{\alpha\beta1}$
x_{α}	Activation gate of I_{α}
y	Activation gate of I_{h}
ω	Activation gate of Force
b_{∞}	Steady-state of b
c_{∞}	Steady-state of c
d_{∞}	Steady-state of d
f_{∞}	Steady-state of f_1 and f_2
g_{∞}	Steady-state of g
h_{∞}	Steady-state of h
k_{∞}	Steady-state of k_1 and k_2
m_{∞}	Steady-state of m
p_{∞}	Steady-state of p
q_{∞}	Steady-state of q
r_{∞}	Steady-state of r_1 and r_2
s_{∞}	Steady-state of s
x_{∞}	Steady-state of x
y_{∞}	Steady-state of y
ω_{∞}	Steady-state of ω
$SS_{\alpha\beta1}$	Steady-state of $x_{\alpha\beta1}$
SS_{α}	Steady-state of x_{α}
$V_{0.5,\alpha\beta1}$	Half activation of $x_{\alpha\beta1}$
$V_{0.5,\alpha}$	Half activation of x_{α}
$z_{\alpha\beta1}$	Gating charge of $x_{\alpha\beta1}$
z_{α}	Gating charge of x_{α}
$\tau_{\alpha\beta1}$	Time constant of $x_{\alpha\beta1}$
τ_{α}	Time constant of x_{α}
τ_b	Time constant of b
τ_c	Time constant of c
τ_d	Time constant of d
τ_{f1}	Time constant of f_1
τ_{f2}	Time constant of f_2
τ_g	Time constant of g
τ_h	Time constant of h
τ_{k1}	Time constant of k_1
τ_{k2}	Time constant of k_2
τ_m	Time constant of m
τ_p	Time constant of p
τ_q	Time constant of q
τ_{r1}	Time constant of r_1
τ_{r2}	Time constant of r_2
τ_s	Time constant of s

Continued on next page

Table S1 – continued from previous page

**Symbols**	**meaning**
τ_x	Time constant of x
τ_y	Time constant of y
τ_ω	Time constant of ω
α_y	Rate constant of activation of y
β_y	Rate constant of deactivation of y
