# Supplementary material for: A Computational Model of the Ionic Currents, Ca2+ Dynamics and Action Potentials Underlying Contraction of Isolated Uterine Smooth Muscle
Source: PLoS One. 2011 Apr 29;6(4):e18685. doi: 10.1371/journal.pone.0018685 (PMC3084699; doi:10.1371/journal.pone.0018685)
Supplement: Table S2 — Definition of gating variables for individual currents and force, and the corresponding experimental temperature and species. (PDF) [file pone.0018685.s010.pdf]

Table S2. Definition of gating variables for individual currents and force, and the corresponding experimental temperature and species. For the activation variables, the superscript refers to the numerical power. For inactivation variables, subscripts 1 and 2 indicate, respectively, fast and slow inactivation. RT – room temperature

**Currents & Force**	**Activation**	**Inactivation**	**Temperature**	**Species**
I_{Na}	m^3	h	$20-22\,°\mathrm{C}$	Rat, human
I_{CaL}	d^2	f_1, f_2	$30-35\,°\mathrm{C}$	Rat
I_{CaT}	b^2	g	$20-24\,°\mathrm{C}$	Rat protein, HEK expression system
I_{K1}	q^2	r_1, r_2	$20-22\,°\mathrm{C}$	Rat, Human
I_{K2}	p^2	k_1, k_2	$20-22\,°\mathrm{C}$	Rat, Human
I_{Ka}	s	x	$20-22\,°\mathrm{C}$	Rat, Human
I_{α}	x_{α}		$20-24\,°\mathrm{C}$	Mammalian protein, *Xenopus laevis* expression system
$I_{\alpha\beta1}$	$x_{\alpha\beta1}$		$20-24\,°\mathrm{C}$	Mammalian protein, *Xenopus laevis* expression system
I_{b}				
I_{h}	y		$22-24, 30\,°\mathrm{C}$	Rat
$I_{\mathrm{Cl(Ca)}}$	c		$35\,°\mathrm{C}$	Rat
I_{NSCC}			RT	Rat
I_{NaK}			$26-36\,°\mathrm{C}$	rodent myocardial and vascular smooth muscle cells
I_{NaCa}			$30-35\,°\mathrm{C}$	Rat
Force	ω		$20-22, 30-33\,°\mathrm{C}$	Rat
