# Supplementary material for: A Computational Model of the Ionic Currents, Ca2+ Dynamics and Action Potentials Underlying Contraction of Isolated Uterine Smooth Muscle
Source: PLoS One. 2011 Apr 29;6(4):e18685. doi: 10.1371/journal.pone.0018685 (PMC3084699; doi:10.1371/journal.pone.0018685)
Supplement: Table S3 — Initial values of the dynamics variables used in model simulations. (PDF) [file pone.0018685.s011.pdf]

Table S3. Initial values of the dynamics variables used in model simulations.

Variables	Initial conditions
V	-53.999
$[Ca^{2+}]_i$	0.000116
m	0.1242
h	0.4073
b	0.5058
g	0.0361
d	0.0102
f_1	0.9077
f_2	0.9077
q	0.2055
r_1	0.1933
r_2	0.1933
p	0.1169
k_1	0.9968
k_2	0.9968
x_α	0.000356
$x_{\alpha\beta1}$	0.002213
s	0.0304
x	0.0888
y	0.002632
c	0.000370
ω	0.2344
