# Supplementary material for: A Computational Model of the Ionic Currents, Ca2+ Dynamics and Action Potentials Underlying Contraction of Isolated Uterine Smooth Muscle
Source: PLoS One. 2011 Apr 29;6(4):e18685. doi: 10.1371/journal.pone.0018685 (PMC3084699; doi:10.1371/journal.pone.0018685)
Supplement: Table S4 — Constant parameter values used in model simulations. (PDF) [file pone.0018685.s012.pdf]

Table S4. Constant parameter values used in model simulations.

Parameters	values
T	308 K
F	96485 $C\,mol^{-1}$
R	8.314 $J\,K^{-1}\,mol^{-1}$
z_{Na}	1
z_K	1
z_{Ca}	2
$[Na^+]_i$	4 mM
$[Na^+]_o$	130 mM
$[K^+]_i$	140 mM
$[K^+]_o$	6 mM
$[Cl^-]_i$	46 mM
$[Cl^-]_o$	130 mM
$[Ca^{2+}]_o$	2.5 mM
$[Mg^{2+}]_o$	0.5 mM
C_m	1 $\mu F\,cm^{-2}$
A_c/V_c	4 cm^{-1}
β	0.015
$\bar{J}_{\mathrm{PMCA}}$	$3.5e^{-7}$ $mM\,ms^{-1}$
$K_{m,\mathrm{PMCA}}$	0.5 µM
n_{PMCA}	2
$\bar{J}_{\mathrm{NaCa}}$	$3.5e^{-6}$ $mM\,ms^{-1}$
$K_{m,\mathrm{Allo}}$	3 µM
n_{Allo}	4
k_{sat}	0.27
γ	0.35
$K_{m,\mathrm{Nai}}$	30.0 mM
$K_{m,\mathrm{Cai}}$	7 µM
$K_{m,\mathrm{Nao}}$	87.5 mM
$K_{m,\mathrm{Cao}}$	1.3 mM
$\bar{g}_{\mathrm{CaL}}$	0.6 $nS\,pF^{-1}$
E_{CaL}	45 mV
$K_{m,\mathrm{CaL}}$	1 µM
$\bar{g}_{\mathrm{CaT}}$	0.058 $nS\,pF^{-1}$
E_{CaT}	42 mV
$\bar{g}_{\mathrm{Na}}$	$0 - 0.12$ $nS\,pF^{-1}$
$\bar{g}_{\mathrm{h}}$	0.0542 $nS\,pF^{-1}$
$\bar{g}_{\mathrm{K}}$	0.8 $nS\,pF^{-1}$
$\bar{g}_{\mathrm{b}}$	0.004 $nS\,pF^{-1}$
$\bar{g}_{\mathrm{K1}}$	0.65 $\bar{g}_{\mathrm{K}}$
$\bar{g}_{\mathrm{K2}}$	0.04 $\bar{g}_{\mathrm{K}}$
$\bar{g}_{\mathrm{Ka}}$	0.2 $\bar{g}_{\mathrm{K}}$
$\bar{g}_{\mathrm{BK}}$	$\bar{g}_{\mathrm{K}}$
p_a	0.2
p_b	0.1
$\bar{g}_{\mathrm{L}}$	0 $nS\,pF^{-1}$

Continued on next page

Table S4 – continued from previous page

**Parameters**	**values**
$\bar{g}_{\mathrm{NS}}$	$0.0123\,\mathrm{nS\,pF^{-1}}$
$P_{\mathrm{Na}}/P_{\mathrm{K}}$	0.35
$P_{\mathrm{Ca}} : P_{\mathrm{Cs}}$	0.89
$P_{\mathrm{Na}} : P_{\mathrm{Cs}}$	0.9
$P_{\mathrm{K}} : P_{\mathrm{Cs}}$	1.3
$\mathrm{K}_{d,\mathrm{Mg}}$	$0.28\,\mathrm{mM}$
$\bar{g}_{\mathrm{Cl}}$	$0.1875\,\mathrm{nS\,pF^{-1}}$
$\bar{g}_{\mathrm{NaK}}$	$1.7\,\mathrm{nS\,pF^{-1}}$
$\mathrm{K}_{d,\mathrm{K}}$	$2.0\,\mathrm{mM}$
n_{K}	1.5
$\mathrm{K}_{m,\mathrm{Na}}$	$22.0\,\mathrm{mM}$
n_{Na}	2
