# Supplementary material for: A Computational Model of the Ionic Currents, Ca2+ Dynamics and Action Potentials Underlying Contraction of Isolated Uterine Smooth Muscle
Source: PLoS One. 2011 Apr 29;6(4):e18685. doi: 10.1371/journal.pone.0018685 (PMC3084699; doi:10.1371/journal.pone.0018685)
Supplement: Appendix S1 — Equations used in the model simulations. (PDF) [file pone.0018685.s013.pdf]

Appendix S1. Equations used in the model simulations.

L-type Calcium current $- I_{\mathrm{CaL}}$

$$I_{\mathrm{CaL}} = \bar{g}_{\mathrm{CaL}} d^2 f_{Ca} (0.8 f_1 + 0.2 f_2)(V - E_{\mathrm{CaL}}) \tag{10}$$

$$f_{Ca} = \frac{1}{1 + \left(\frac{[\mathrm{Ca}^{2+}]_{\mathrm{i}}}{\mathrm{K}_{d,\mathrm{CaL}}} \right)^4} \tag{11}$$

$$d_\infty = \frac{1}{1 + \exp\left(\frac{-(V+22)}{7} \right)} \tag{12}$$

$$f_\infty = \frac{1}{1 + \exp\left(\frac{V+38}{7} \right)} \tag{13}$$

$$\tau_d = 2.29 + \frac{5.7}{1 + \left(\frac{V + 29.97}{9} \right)^2} \tag{14}$$

$$\tau_{f1} = 12\mathrm{ms} \tag{15}$$

$$\tau_{f2} = 90.97 \left(1 - \frac{1}{\left(1 + \exp\left(\frac{V + 13.96}{45.38} \right) \right) \left(1 + \exp\left(\frac{-(V+9.5)}{3.39} \right) \right)} \right) \tag{16}$$

$$\frac{dd}{dt} = \frac{d_\infty - d}{\tau_d} \tag{17}$$

$$\frac{df_1}{dt} = \frac{f_\infty - f_1}{\tau_{f1}} \tag{18}$$

$$\frac{df_2}{dt} = \frac{f_\infty - f_2}{\tau_{f2}} \tag{19}$$

Sodium current – I_{Na}

$$I_{\mathrm{Na}} = \bar{g}_{\mathrm{Na}} m^3 h (V - E_{\mathrm{Na}}) \tag{20}$$

$$E_{\mathrm{Na}} = \frac{RT}{F} \ln \left(\frac{[\mathrm{Na}^+]_{\mathrm{o}}}{[\mathrm{Na}^+]_{\mathrm{i}}} \right) \tag{21}$$

$$m_\infty = \frac{1}{1 + \exp\left(\frac{-(V + 35.96)}{9.24} \right)} \tag{22}$$

$$h_\infty = \frac{1}{1 + \exp\left(\frac{V + 57}{8} \right)} \tag{23}$$

$$\tau_m = 0.25 + \frac{7}{1 + \exp\left(\frac{V + 38}{10} \right)} \tag{24}$$

$$\tau_h = 0.9 + \frac{1002.85}{1 + \left(\frac{V + 47.5}{1.5} \right)^2} \tag{25}$$

$$\frac{dm}{dt} = \frac{m_\infty - m}{\tau_m} \tag{26}$$

$$\frac{dh}{dt} = \frac{h_\infty - h}{\tau_h} \tag{27}$$

T-type Calcium current – I_{CaT}

$$I_{\mathrm{CaT}} = \bar{g}_{\mathrm{CaT}} b^2 g (V - E_{\mathrm{CaT}}) \tag{28}$$

$$b_\infty = \frac{1}{1 + \exp\left(\dfrac{-(V + 54.23)}{9.88}\right)} \tag{29}$$

$$g_\infty = 0.02 + \frac{0.98}{1 + \exp\left(\dfrac{V + 72.98}{4.64}\right)} \tag{30}$$

$$\tau_b = 0.45 + \frac{3.9}{1 + \left(\dfrac{V + 66}{26}\right)^2} \tag{31}$$

$$\tau_g = \left(150 - \frac{150}{\left(1 + \exp\left(\dfrac{V - 417.43}{203.18}\right)\right)\left(1 + \exp\left(\dfrac{-(V + 61.11)}{8.07}\right)\right)}\right) \tag{32}$$

$$\frac{db}{dt} = \frac{b_\infty - b}{\tau_b} \tag{33}$$

$$\frac{dg}{dt} = \frac{g_\infty - g}{\tau_g} \tag{34}$$

Hyperpolarisation-activated current $-$ I_h

$$I_\mathrm{h} = \bar{g}_\mathrm{h} y (V - E_\mathrm{h}) \tag{35}$$

$$E_\mathrm{h} = \frac{RT}{F} \ln\left(\frac{[\mathrm{K}^+]_\mathrm{o} + (P_\mathrm{Na}/P_\mathrm{K})[\mathrm{Na}^+]_\mathrm{o}}{[\mathrm{K}^+]_\mathrm{i} + (P_\mathrm{Na}/P_\mathrm{K})[\mathrm{Na}^+]_\mathrm{i}}\right) \tag{36}$$

$$y_\infty = \frac{1}{1 + \exp\left(\dfrac{V + 105.39}{8.66}\right)} \tag{37}$$

$$\tau_y = \frac{1}{3.5e^{-6}\exp(-0.0497V) + 0.04\exp(0.0521V)} \tag{38}$$

$$\frac{dy}{dt} = \frac{y_\infty - y}{\tau_y} \tag{39}$$

Voltage dependent potassium current – I_{K1}

$$I_{\mathrm{K1}} = \bar{g}_{\mathrm{K1}} q^2 (0.38 r_1 + 0.62 r_2)(V - E_{\mathrm{K}}) \tag{40}$$

$$E_{\mathrm{K}} = \frac{RT}{F} \ln\left(\frac{[\mathrm{K}^+]_{\mathrm{o}}}{[\mathrm{K}^+]_{\mathrm{i}}}\right) \tag{41}$$

$$q_\infty = \frac{1}{1 + \exp\left(\frac{-(V + 18.67)}{26.66}\right)} \tag{42}$$

$$r_\infty = \frac{1}{1 + \exp\left(\frac{V + 63}{6.3}\right)} \tag{43}$$

$$\tau_q = \frac{500}{1 + \left(\frac{V + 60.71}{15.79}\right)^2} \tag{44}$$

$$\tau_{r1} = \frac{5e^4}{1 + \left(\frac{V + 62.71}{35.86}\right)^2} \tag{45}$$

$$\tau_{r2} = 3e^4 + \frac{2.2e^5}{1 + \exp\left(\frac{V + 22}{4}\right)} \tag{46}$$

$$\frac{dq}{dt} = \frac{q_\infty - q}{\tau_q} \tag{47}$$

$$\frac{dr_1}{dt} = \frac{r_\infty - r_1}{\tau_{r1}} \tag{48}$$

$$\frac{dr_2}{dt} = \frac{r_\infty - r_2}{\tau_{r2}} \tag{49}$$

Voltage dependent potassium current – I_{K2}

$$I_{\mathrm{K2}} = \bar{g}_{\mathrm{K2}} p^2 (0.75 k_1 + 0.25 k_2)(V - E_{\mathrm{K}}) \tag{50}$$

$$p_\infty = \frac{1}{1 + \exp\left(\dfrac{-(V + 0.948)}{17.91}\right)} \tag{51}$$

$$k_\infty = \frac{1}{1 + \exp\left(\dfrac{(V + 21.2)}{5.7}\right)} \tag{52}$$

$$\tau_p = \frac{100}{1 + \left(\dfrac{V + 64.1}{28.67}\right)^2} \tag{53}$$

$$\tau_{k1} = 1e^6 \left(1 - \frac{1}{\left(1 + \exp\left(\dfrac{V - 315}{50}\right)\right)\left(1 + \exp\left(\dfrac{-(V + 74.9)}{8}\right)\right)}\right) \tag{54}$$

$$\tau_{k2} = 2.5e^6 \left(1 - \frac{1}{\left(1 + \exp\left(\dfrac{V - 132.87}{25.40}\right)\right)\left(1 + \exp\left(\dfrac{-(V + 24.92)}{2.68}\right)\right)}\right) \tag{55}$$

$$\frac{dp}{dt} = \frac{p_\infty - p}{\tau_p} \tag{56}$$

$$\frac{dk_1}{dt} = \frac{k_\infty - k_1}{\tau_{k1}} \tag{57}$$

$$\frac{dk_2}{dt} = \frac{k_\infty - k_2}{\tau_{k2}} \tag{58}$$

Transient potassium current – I_{Ka}

$$I_{\mathrm{Ka}} = \bar{g}_{\mathrm{Ka}} s x (V - E_{\mathrm{K}}) \tag{59}$$

$$s_\infty = \frac{1}{1 + \exp\left(\frac{-(V + 27.79)}{7.57}\right)} \tag{60}$$

$$x_\infty = 0.02 + \frac{0.98}{1 + \exp\left(\frac{V + 69.5}{6}\right)} \tag{61}$$

$$\tau_s = \frac{17}{1 + \left(\frac{V + 20.52}{35}\right)^2} \tag{62}$$

$$\tau_x = 7.5 + \frac{10}{1 + \left(\frac{V + 34.18}{120}\right)^2} \tag{63}$$

$$\frac{ds}{dt} = \frac{s_\infty - s}{\tau_s} \tag{64}$$

$$\frac{dx}{dt} = \frac{x_\infty - x}{\tau_x} \tag{65}$$

Calcium-activation potassium current – $I_{\mathrm{K,Ca}}$

$$I_{\mathrm{K(Ca)}} = \bar{g}_{\mathrm{K(Ca)}}(p_a I_\alpha + p_b I_{\alpha\beta1}) \tag{66}$$

$$I_\alpha = x_\alpha(V - E_{\mathrm{K}}) \tag{67}$$

$$z_\alpha = \frac{8.38}{1 + \left(\frac{1000[\mathrm{Ca}^{2+}]_\mathrm{i} + 1538.29}{739.06}\right)^2} - \frac{0.749}{1 + \left(\frac{1000[\mathrm{Ca}^{2+}]_\mathrm{i} - 0.063}{0.162}\right)^2} \tag{68}$$

$$V_{0.5,\alpha} = \frac{5011.47}{1 + \left(\frac{1000[\mathrm{Ca}^{2+}]_\mathrm{i} + 0.238}{0.000239}\right)^{0.423}} - 37.51 \tag{69}$$

$$SS_\alpha = \frac{1}{1 + \exp\left(-\frac{z_\alpha F(V - V_{0.5,\alpha})}{RT}\right)} \tag{70}$$

$$\tau_\alpha = \frac{2.41}{1 + \left(\frac{V - 158.78}{-52.15}\right)^2} \tag{71}$$

$$\frac{dx_\alpha}{dt} = \frac{SS_\alpha - x_\alpha}{\tau_\alpha} \tag{72}$$

$$I_{\alpha\beta1} = x_{\alpha\beta1}(V - E_{\mathrm{K}}) \tag{73}$$

$$z_{\alpha\beta1} = \frac{1.4}{1 + \left(\frac{1000[\mathrm{Ca}^{2+}]_\mathrm{i} + 228.71}{684.95}\right)^2} - \frac{0.681}{1 + \left(\frac{1000[\mathrm{Ca}^{2+}]_\mathrm{i} - 0.219}{0.428}\right)^2} \tag{74}$$

$$V_{0.5,\alpha\beta1} = \frac{8540.23}{1 + \left(\frac{1000[\mathrm{Ca}^{2+}]_\mathrm{i} + 0.401}{0.00399}\right)^{0.668}} - 109.28 \tag{75}$$

$$SS_{\alpha\beta1} = \frac{1}{1 + \exp\left(-\frac{z_{\alpha\beta1} F(V - V_{0.5,\alpha\beta1})}{RT}\right)} \tag{76}$$

$$\tau_{\alpha\beta1} = \frac{13.8}{1 + \left(\frac{V - 153.02}{66.5}\right)^2} \tag{77}$$

$$\frac{dx_{\alpha\beta1}}{dt} = \frac{SS_{\alpha\beta1} - x_{\alpha\beta1}}{\tau_{\alpha\beta1}} \tag{78}$$

Background current – I_b

$$I_\mathrm{b} = \bar{g}_\mathrm{b}(V - E_\mathrm{K}) \tag{79}$$

Calcium-activated chloride current – $I_\mathrm{Cl(Ca)}$

$$I_\mathrm{Cl(Ca)} = \bar{g}_\mathrm{Cl} c (V - E_\mathrm{Cl}) \tag{80}$$

$$E_\mathrm{Cl} = \frac{RT}{F} \ln\left(\frac{[\mathrm{Cl}^-]_\mathrm{i}}{[\mathrm{Cl}^-]_\mathrm{o}}\right) \tag{81}$$

$$\mathrm{K}_{1,\mathrm{Cl}} = 0.0006 \exp\left(\frac{2.53FV}{RT}\right) \tag{82}$$

$$\mathrm{K}_{2,\mathrm{Cl}} = 0.1 \exp\left(\frac{-5FV}{RT}\right) \tag{83}$$

$$c_\infty = \frac{1}{1 + \mathrm{K}_{2,\mathrm{Cl}}\left(\left(\frac{\mathrm{K}_{1,\mathrm{Cl}}}{[\mathrm{Ca}^{2+}]_\mathrm{i}}\right)^2 + \frac{\mathrm{K}_{1,\mathrm{Cl}}}{[\mathrm{Ca}^{2+}]_\mathrm{i}} + 1\right)} \tag{84}$$

$$\tau_c = \frac{210}{1 + \exp\left(\frac{V + 4.56}{11.62}\right)} + \frac{170}{1.0 + \exp\left(\frac{-(V + 25.5)}{11.62}\right)} - 160 \tag{85}$$

$$\frac{dc}{dt} = \frac{c_\infty - c}{\tau_c} \tag{86}$$

Non-selective cation current – I_{NSCC}

$$I_{\text{NSCC}} = g_{NS} f_{Mg} (V - E_{\text{NS}}) \tag{87}$$

$$g_{NS} = \bar{g}_{\text{NS}} \left[0.5g([\text{Ca}^{2+}]_{\text{o}}) + g([\text{Na}^{+}]_{\text{o}}) + 1.19g([\text{K}^{+}]_{\text{o}})\right] + \bar{g}_{\text{L}} \tag{88}$$

$$g([\text{X}]_{\text{o}}) = \frac{1}{g_s} \frac{0.03}{1 + \left(\dfrac{150}{[\text{X}]_{\text{o}} + 10^{-8}}\right)^2}, \quad g_s = \begin{cases} 0.000525 & \text{if ion is Ca}^{2+} \\ 0.0123 & \text{otherwise} \end{cases} \tag{89}$$

$$f_{Mg} = 0.1 + \frac{0.9}{1 + \left(\dfrac{[\text{Mg}^{2+}]_{\text{o}}}{\text{K}_{d,\text{Mg}}}\right)^{1.3}} \tag{90}$$

$$E_{\text{NS}} = \frac{RT}{F} \ln \left(\frac{\dfrac{P_{\text{Na}}}{P_{\text{Cs}}}[\text{Na}^{+}]_{\text{o}} + \dfrac{P_{\text{K}}}{P_{\text{Cs}}}[\text{K}^{+}]_{\text{o}} + \dfrac{4P'_{\text{Ca}}}{P_{\text{Cs}}}[\text{Ca}^{2+}]_{\text{o}}}{\dfrac{P_{\text{Na}}}{P_{\text{Cs}}}[\text{Na}^{+}]_{\text{i}} + \dfrac{P_{\text{K}}}{P_{\text{Cs}}}[\text{K}^{+}]_{\text{i}} + \dfrac{4P'_{\text{Ca}}}{P_{\text{Cs}}}[\text{Ca}^{2+}]_{\text{i}}} \right) \tag{91}$$

$$P'_{\text{Ca}} = \frac{P_{\text{Ca}}}{1 + \exp\left(\dfrac{VF}{RT}\right)} \tag{92}$$

Sodium potassium pump current – I_{NaK}

$$I_{\mathrm{NaK}} = \bar{g}_{\mathrm{NaK}}\, f_{NaK}\, k_{NaK}\, n_{NaK} \tag{93}$$

$$f_{NaK} = \frac{1}{\left(\begin{array}{c} 1 + 0.125\exp\left(\dfrac{-0.1VF}{RT}\right) + \\ 0.00219\exp\left(\dfrac{[\mathrm{Na}^+]_{\mathrm{o}}}{49.71}\right)\exp\left(\dfrac{-1.9VF}{RT}\right)\end{array}\right)} \tag{94}$$

$$k_{NaK} = \frac{1}{1 + \left(\dfrac{\mathrm{K}_{m,\mathrm{K}}}{[\mathrm{K}^+]_{\mathrm{o}}}\right)^{n_{\mathrm{K}}}} \tag{95}$$

$$n_{NaK} = \frac{1}{1 + \left(\dfrac{\mathrm{K}_{m,\mathrm{Na}}}{[\mathrm{Na}^+]_{\mathrm{i}}}\right)^{n_{\mathrm{Na}}}} \tag{96}$$

$[\mathrm{Ca}^{2+}]_{\mathrm{i}}$ **dynamics**

$$\frac{d[\mathrm{Ca}^{2+}]_{\mathrm{i}}}{dt} = -(J_{\mathrm{Ca,mem}} + J_{\mathrm{NaCa}} + J_{\mathrm{PMCA}}) \tag{97}$$

Ca^{2+} **flux from plasma membrane** Ca^{2+}**-ATPase** – J_{PMCA}

$$J_{\mathrm{PMCA}} = \frac{\bar{J}_{\mathrm{PMCA}}}{1 + \left(\dfrac{\mathrm{K}_{m,\mathrm{PMCA}}}{[\mathrm{Ca}^{2+}]_{\mathrm{i}}}\right)^{n_{\mathrm{PMCA}}}} \tag{98}$$

Ca^{2+} **flux from sodium calcium exchanger** – J_{NaCa}

$$J_{\mathrm{NaCa}} = \frac{\bar{J}_{\mathrm{NaCa}}\ f_{allo}\left([\mathrm{Na}^+]_\mathrm{i}^3[\mathrm{Ca}^{2+}]_\mathrm{o} f_{2,NaCa} - [\mathrm{Na}^+]_\mathrm{o}^3[\mathrm{Ca}^{2+}]_\mathrm{i} f_{1,NaCa}\right)}{(1+k_{sat} f_{1,NaCa})\left(\begin{array}{c}\mathrm{K}_{m,\mathrm{Cao}}[\mathrm{Na}^+]_\mathrm{i}^3 + \mathrm{K}_{m,\mathrm{Nao}}^3[\mathrm{Ca}^{2+}]_\mathrm{i} + \\ [\mathrm{Ca}^{2+}]_\mathrm{o}[\mathrm{Na}^+]_\mathrm{i}^3 + [\mathrm{Na}^+]_\mathrm{o}^3[\mathrm{Ca}^{2+}]_\mathrm{i} + \\ \mathrm{K}_{m,\mathrm{Nai}}^3[\mathrm{Ca}^{2+}]_\mathrm{o}\left(1+\dfrac{[\mathrm{Ca}^{2+}]_\mathrm{i}}{\mathrm{K}_{m,\mathrm{Cai}}}\right) + \\ [\mathrm{Na}^+]_\mathrm{o}^3\mathrm{K}_{m,\mathrm{Cai}}\left(1+\left(\dfrac{[\mathrm{Na}^+]_\mathrm{i}}{\mathrm{K}_{m,\mathrm{Nai}}}\right)^3\right)\end{array}\right)} \quad (99)$$

$$f_{1,NaCa} = \exp\left((\gamma-1)\frac{VF}{RT}\right) \quad (100)$$

$$f_{2,NaCa} = \exp\left(\gamma\frac{VF}{RT}\right) \quad (101)$$

$$f_{allo} = \frac{1}{1+\left(\dfrac{\mathrm{K}_{m,\mathrm{Allo}}}{[\mathrm{Ca}^{2+}]_\mathrm{i}}\right)^{n_{\mathrm{Allo}}}} \quad (102)$$

$$I_{\mathrm{NaCa}} = \frac{z_{Ca} F V_c}{C_m A_c \beta} J_{\mathrm{NaCa}} \quad (103)$$

$[\mathrm{Ca}^{2+}]_\mathrm{i}$**-dependent force**

$$\omega_\infty = \frac{1}{1+\left(\dfrac{\mathrm{K}_{m,\mathrm{F}}}{[\mathrm{Ca}^{2+}]_\mathrm{i}}\right)^{n_\mathrm{F}}} \quad (104)$$

$$\tau_\omega = 4000\left(0.235 + \frac{1-0.235}{1+\left(\dfrac{[\mathrm{Ca}^{2+}]_\mathrm{i}}{\mathrm{K}_{m,\mathrm{F}}}\right)^{n_\mathrm{F}}}\right) \quad (105)$$
